# Beyond Heuristics: A Model-Agnostic Framework for Uncertainty Quantification in QSAR via Adaptive Conformal Prediction

**DOI:** 10.1021/acs.chemrestox.6c00065

**Published:** 2026-06-22

**Authors:** Nina Jeliazkova, Nikolay Kochev, Luchesar Iliev, Vedrin Jeliazkov

**Affiliations:** † 617674 Ideaconsult Ltd., Sofia 1000, Bulgaria; ‡ University of Plovdiv, Plovdiv 4000, Bulgaria

## Abstract

Reliable quantification of uncertainty is critical for the interpretation and regulatory use of the QSAR models. Applicability domain (AD) assessment was introduced precisely for this purpose—the original OECD guidance defines AD in terms of prediction reliability—yet in practice AD metrics output heuristic similarity scores without statistically guaranteed confidence estimates. We present conformal prediction as a calibration layer that retrofits any QSAR models into a confidence predictor, producing prediction intervals for regression and prediction sets for classification at a user-specified nominal confidence level (e.g., 90%), with statistically guaranteed coverage, without retraining, using only model predictions and a calibration set. The guarantee holds under the exchangeability assumption—that calibration and test compounds are drawn from the same input space—and follows as a mathematical consequence of the rank-based calibration procedure. When the assumption is violated, coverage may fall below the nominal level—signaled by widening intervals and shrinking singleton rates. The framework uses auxiliary models trained on molecular fingerprints as nonconformity scores, a role that most existing AD indices can equally fulfill; a novel ordinal distance strategy extends the approach to hard-label classifiers by generating pseudoproabilities compatible with standard conformal methods. Applied to over 100 VEGA QSAR models spanning physicochemical properties, toxicity, and environmental endpoints, the framework consistently achieves nominal coverage across all models and endpoint types. Conformal efficiency metrics—prediction interval width for regression and singleton rate for classification—correlate strongly with AD indices, demonstrating that CP formalizes and quantifies what AD heuristics approximate: the relationship between structural novelty and prediction reliability, successfully transforming heuristic chemical similarity into statistically valid prediction intervals or label sets. Large-scale application to the EPA CompTox chemical inventory demonstrates practical deployment at a regulatory scale. An open-source pipeline facilitates application to any QSAR/QSPR platform, enabling an improved transparency and reliability assessment.

## Graphical abstract

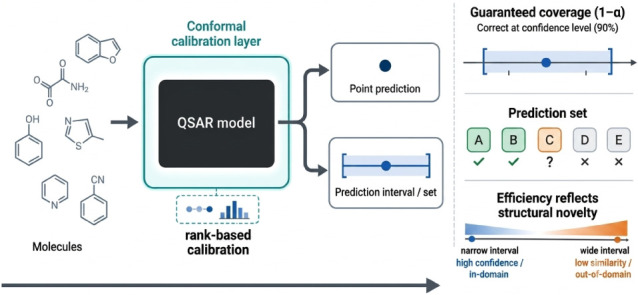

## 1 Introduction

QSAR/QSPR models face a longstanding challenge: the quantification of prediction uncertainty. Regulatory frameworks require not only point predictions but also transparent assessments of their reliability. The OECD Guidance on the validation of (Q)­SAR^1^ states “the domain of application for a (Q)­SAR model describes whether the model will predict an endpoint for a specific chemical with a given reliability”. The concept of applicability domain was introduced as both the chemical structure and response space in which the model makes predictions with a given reliability.^2^ Chemical structure space was recommended as a proxy for assessing the reliability of QSAR predictions using applicability domain (AD) methods^2^—the assumption being that structural similarity to training compounds implies reliable prediction. This assumption is reasonable but not formally guaranteed—a compound may lie well within the training domain yet be poorly predicted due to activity cliffs, noise in the training data, or model overfitting. Conversely, a structurally novel compound may be predicted accurately if the model has captured the relevant mechanistic features. The response space is also part of the recommendation^2^ and subsequently adopted in different approaches—response ranges, joint input-response distribution,^3^ and distance-to-model.^4,5^ Distance-to-model approaches in QSAR define a scalar measure intended to capture the notion of how “familiar” a query compound is with respect to the training domain. Depending on the specific formulation, this measure may be based on structural similarity, model-space behavior, or response correlations, but in all cases it serves as a proxy for extrapolation risk rather than a direct estimate of predictive error. In likelihood-based extensions, this empirical relationship is further modeled by estimating a conditional error distribution given the distance-to-model, typically using parametric or mixture models. While this approach provides a structured way to translate distance into uncertainty, it remains dependent on distributional assumptions (e.g., bimodal Gaussian error model^4^) and does not provide statistical reliability guarantees. In many tools, AD is interpreted in binary terms—predictions are either accepted as “inside” or flagged as “outside” the domain. The VEGA QSAR platform^6^ extends this concept by introducing an applicability domain index (ADI),^7^ a continuous score ranging from 0 to 1 that combines multiple diagnostics, such as similarity to the training set, descriptor range checks, and structural alerts. This provides a more nuanced assessment than a simple binary flag. Nevertheless, ADI and other domain metrics remain heuristic: they depend on how descriptors and similarity thresholds are defined, and different implementations may yield different reliability scores for the same compound, which are not comparable objectively. More importantly, none of the applicability domain metrics available provides a formal statistical guarantee of how the true value is related to the estimated AD value. The limitations of distance-based and similarity-weighted applicability domain indices have been debated since their inception over two decades ago.^2,3,8^ While all early methods were essential for practical reasons, they remain^7^ fundamentally heuristic, often failing to provide a consistent correlation between applicability domain metrics and actual predictive reliability. This study represents a natural evolution of that philosophy: moving beyond binary and heuristic domain classifications toward a mathematically grounded framework where uncertainty is expressed as a calibrated interval rather than a qualitative category. For regulatory applications and early-stage Safe and Sustainable by Design (SSbD)^9^ decisions, where transparency in uncertainty is critical, there is a need for methods that provide calibrated, quantitative confidence estimates and statistical guarantees that the true value lies within a specified error tolerance.

Conformal prediction (CP) is a statistical framework^10–13^ that can be applied on top of any machine learning model (thus, model-agnostic). CP addresses the same underlying need as the original QSAR applicability domain definition—quantifying prediction reliability in relation to the input and response space of the model—while offering mathematical guarantees: under mild assumptions of exchangeability, the true value will fall within the prediction region at the chosen confidence level α. CP produces different output types depending on the prediction task. For regression endpoints, the output is a **prediction interval** (Figure 1 top); for classification endpoints, the output is a **prediction set**—the subset of classes that cannot be rejected at level α. In both cases, the coverage guarantee means that the true response falls inside the interval or the set with probability ≥1 – α. The **coverage rate** is the primary validity criterion (Figure 1 bottom). Per molecule, the coverage is binary (the true value is in or out of the interval) (Figure 1 top). Aggregated over the test set, the mean coverage rate must satisfy coverage ≥1 – α; a failure indicates a distribution shift between calibration and test sets. This guarantee holds on average over all test molecules, not per molecule.

**Figure 1:**
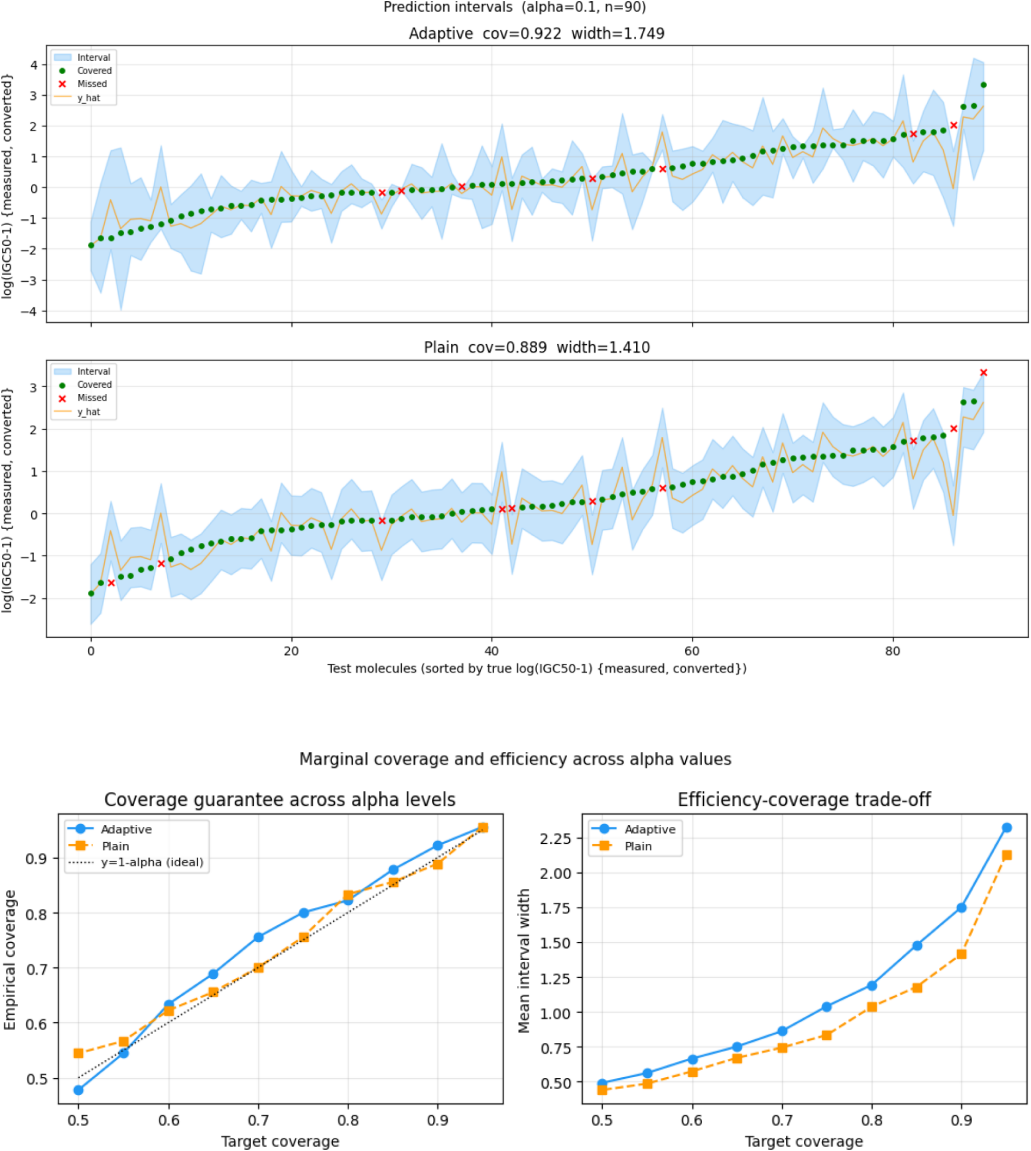
Conformal prediction trade-offs between calibration and interval efficiency—marginal coverage and efficiency across multiple confidence levels α. The adaptive approach consistently provided coverage closer to the theoretical 1−α guarantee, whereas the plain conformal method produced narrower but slightly under-covering intervals. Comparison of adaptive and plain conformal prediction intervals for the log­(IC50–1) endpoint. Bottom panels show prediction intervals for individual test compounds at a significance level α = 0.1 (target coverage 90%). Test compounds are sorted by measured endpoint values. The orange line represents predicted values, shaded blue regions indicate conformal prediction intervals, green circles denote covered observations, and red crosses indicate observations outside the interval. Data and model predictions from reference 4 and ochem.eu.

The **efficiency** measures the informativeness of the output. For regression, efficiency is the mean interval width; for classification, it is the mean prediction set size. The calibration objective is to minimize the width (set size) subject to coverage ≥1 – α. There is a fundamental trade-off between coverage and efficiency: increasing interval width (or expanding prediction sets) improves the likelihood of capturing the true value, but reduces practical usefulness (Figure 1 bottom).

Conformal prediction proceeds in three steps. First, a **nonconformity measure or score** *s*(**x** *_i_*,*y*_*i*_) is computed for each molecule *i* in a held-out calibration set of size *n*. The **score** quantifies how unusual is the pair (**x**_*i*_,*y*_*i*_) relative to the model prediction; i.e., how poorly the model accounts for the true response at molecule *i.* Higher scores indicate unusual (less reliable) predictions, while lower scores indicate predictions that fit well with previous data. For the plain variant, the default choice is *s* = |*y* – *ŷ*|; for the adaptive variant, *s* = |*y* – *ŷ*|/σ̂(**x**), where σ̂(**x**) is a molecule-specific uncertainty estimate.

The second step is to compute a single **calibration quantile** per model, using the nonconformity **scores of the calibration data set** that was not used during the first step:

$$\mathrm{q̂}=\mathrm{Quantile}\left(\left\{s_{1},...,s_{n}\right\},\frac{\left\lceil \left(n+1\right)\left(1-\alpha \right)\right\rceil }{n}\right)$$

where ⌈·⌉ denotes the ceiling function (rounding up to the nearest integer), which ensures the selection of the smallest quantile threshold that guarantees coverage of at least 1 – α.

Finally, at inference time, a **prediction interval** *C*(*x*) is constructed for each new molecule: *C*(*x*) = *ŷ* ± *q̂* for the plain variant, and *C*(*x*) = *ŷ* ± *q̂*·σ̂(*x*) for the adaptive variant. Plain intervals have identical width for every molecule; adaptive intervals are molecule-specific, narrow where the model is confident and wide where it is not (Figure 1 bottom). For classification, the score is typically: s = 1 – *p̂*(*y*|*x*) (the complement of the probability assigned to the true class). A confidently wrong prediction receives a score near 1; a confident correct prediction receives a score near 0. The calibration quantile *q̂* then defines a probability threshold: at inference time, the **prediction set consists of all class labels** *k* for which 1 – *p̂*(*k*|*x*) ≤ *q̂*, or equivalently, all classes assigned probability ≥1–*q̂*. Given a classification problems with labels *low*, *middle*, and *high*, CP outputs a prediction set that may contain zero, one, or multiple labels. Examples include the empty set {}, {high}, or {*middle*, *high*}. The prediction set shows how certain the model is: one label (a singleton) means a clear prediction, multiple labels mean uncertainty between options, and an empty set means the model could not make a reliable prediction. When class imbalance is substantial, Mondrian CP^10^ is recommended to perform calibration by class label, computing a separate quantile per class and providing per-class coverage guarantees.

Importantly, CP does not restrict the selection of a nonconformity score; any function that provides *s*(**x** *_i_*,*y*_*i*_) values proportional to prediction errors is a valid choice. The plain absolute residuals/true class probability is the default choice for regression/classification, respectively; the adaptive score generally improves efficiency, but requires an auxiliary estimate σ̂(**x**). These can be approximated from ensemble variance (if available from the original model), or using approaches similar to applicability domain—e.g., k-nearest-neighbor distances in feature space or leverage—that are straightforward to compute but are not necessarily monotonically correlated with residuals. A more direct alternative is to train a dedicated auxiliary model that predicts |*y* – *ŷ*| from molecular descriptors, explicitly targeting the local error scale. This approach yields better-calibrated σ̂(*x*) values when sufficient data are available, but introduces an additional modeling step.

Existing AD indices, including distance-to-model (DM) metrics^4,5^ (similarity-based or ensemble-based, standard deviation across predictions, or likelihood-based), can be understood as nonconformity measures in the CP framework: they quantify how unusual a query compound is relative to the training distribution and can serve directly as σ̂(*x*) in the adaptive score. Ensemble standard deviation in particular is the nonconformity measure underlying cross-conformal prediction, where leave-one-out or cross-validated residuals implicitly encode model disagreement. Likelihood-based DM methods go further by explicitly modeling the conditional error distribution p­(e|x) as a parametric function of distance—typically a Gaussian or Gaussian mixture—which provides a richer uncertainty estimate but requires distributional assumptions and a stable error-distance relationship across chemical space, conditions that may not hold in high-dimensional or sparsely populated descriptor spaces. CP avoids explicit modeling of this relationship altogether, using rank-based calibration of residuals and can be interpreted as a calibration layer on top of AD measures, converting any such measure into prediction intervals with finite-sample coverage guarantees.

Quantifying predictive uncertainty in QSAR has been an active research topic for over a decade and is mostly considered complementary to the applicability domain.^14–17^ Several methods exist to quantify predictive uncertainty in machine learning models, each with distinct strengths and limitations. Bayesian approaches offer rich posterior uncertainty but typically require model retraining and provide probabilistic rather than strict frequentist guarantees. Quantile regression allows prediction of conditional quantiles and handles heteroscedasticity, but interval validity is not formally guaranteed and retraining is usually needed. Bootstrap and resampling methods are model-agnostic and can be applied post hoc in some cases, yet finite-sample coverage is approximate and computational costs can be high. Ensemble approaches capture model uncertainty and improve accuracy but generally require multiple model training and lack formal coverage guarantees. Probability calibration techniques^18^ can adjust predicted probabilities posthoc but do not provide prediction intervals. Compared to existing uncertainty quantification methods, conformal prediction is distinctive in being model-agnostic, distribution-free, and the only approach that provides a finite-sample marginal coverage guarantee without retraining the primary predictor—valid prediction intervals follow from any underlying model provided the calibration and test data sets are exchangeable. CP guarantees marginal coverage, not per-point certainty, although there is new research on providing individual guarantees.^19^

CP is model-agnostic in the sense established in the machine learning literature:^20^ it treats the underlying QSAR model as a black box, requiring only its predictions and a held-out calibration set, and can be applied to any model architecture (e.g., random forest, neural network, gradient boosting), without access to internal parameters, descriptors, or training details. This is the sense in which widely adopted implementations such as MAPIE (Model Agnostic Prediction Interval Estimator^21^) use the term. The same definition is used in recent CP literature.^22–24^ This is distinct from claiming that uncertainty estimates are independent of the model and model quality, which would be impossible; the width and calibration of CP intervals directly reflect the residual behavior of the underlying predictor. Importantly, model-agnosticism does not conflict with the OECD guidance that the applicability domain should be defined in a model-specific manner: CP intervals are derived from the outputs of a specific model on its own calibration set and are therefore implicitly tied to that model’s descriptors and statistical approach. What the framework adds is that uncertainty quantification can be performed by any party with access to model predictions and calibration data, without requiring the full model internals—extending rigorous uncertainty quantification beyond the original model developer to model users.

Although CP has been explored in cheminformatics and drug discovery,^11,25–28^ systematic applications across large QSAR model suites and regulatory inventories remain limited. In particular, no study has evaluated CP across the breadth of endpoints covered by existing predictive platforms. Within this work, we developed a generic methodology to quantify the uncertainty of existing regression and classification QSAR models (including ones with hard class labels) through conformal prediction. The methodology is demonstrated through the uncertainty assessment of VEGA^6^ models, starting from VEGA training and test data sets, and training auxiliary models using chemical structures to enable adaptive conformal predictions; the underlying VEGA models were not retrained. CP statistics (coverage, efficiency, exchangeability) were collected on the VEGA test sets. Subsequently, the VEGA models and the trained auxiliary models were applied to a large external data set retrieved from the EPA CompTox dashboard^29^ to evaluate performance on an independent chemical inventory. No models from the EPA CompTox dashboard were used in this study. It should be noted that the VEGA platform includes reimplementations of models originally developed in other frameworks, including OPERA;^30^ throughout this work, we treat all models as VEGA models without distinction, as our methodology operates on model predictions and does not depend on the underlying model provenance. VEGA models are selected as a showcase because of being available as a standalone downloadable software (important to handle confidential structures in the SSbD context); being recommended by the PARC SSbD Toolbox;^31^ and providing a composite quantitative applicability domain index (ADI) that allows comparison to the conformal prediction efficiency metrics. We report strong Spearman rank correlations of conformal prediction efficiency (e.g., interval width, singleton predictions rate) to ADI in both regression and classification models. To demonstrate that the methodology is not restricted to VEGA models, a general-purpose tutorial pipeline was provided, accepting any data sets with SMILES, predicted and experimental values, and optional applicability domain indices (for comparison). The pipelines (the full pipeline used in this study and the tutorial) are available under an open-source license at https://github.com/ideaconsult/qubounds.

## 2 Methods

This section demonstrates that conformal prediction can transform any classification or regression (Q)­SAR into a confidence predictor with a user-defined confidence level (e.g., 90% for α = 0.1). For regression models, the output is a numeric range that is guaranteed to contain (cover) the true experimental value for at least 90% of test compounds. Narrower intervals indicate higher model confidence for the specific compound, while wider intervals indicate higher model error. For classification endpoints—e.g., mutagenicity, biodegradability, skin sensitization—the output is a prediction set (subset of class labels): also guaranteed to contain the true experimental value for at least 90% of test compounds. For a three-class endpoint such as skin sensitization (*Nonsensitizer*, *Weak sensitizer*, *Strong sensitizer*), a singleton set {*Weak sensitizer*} is an unambiguous result; a two-class set {*Weak sensitizer, Strong sensitizer*} indicates the model cannot distinguish between these outcomes at the requested confidence, but is still a meaningful result (sensitizer); a full three-class set indicates an unreliable prediction. An empty set is a valid outcome under the LAC algorithm, essentially indicating that the compound is unlike anything in the calibration set.

The nonconformity score is the only modeling choice that affects the practical utility of CP outputs: coverage is guaranteed regardless, but the score determines how narrow the intervals or prediction sets will be. Any function of (*x_i_*, *y_i_*) that assigns higher values to less expected observations is a valid nonconformity score—raw residuals, ensemble variance, distance-to-training-set metrics, or learned auxiliary models are all legitimate choices. For the adaptive variant used throughout this study, the score takes the form |*y* – *ŷ*|/σ̂(*x*), requiring an auxiliary model σ̂(*x*) that estimates local residual magnitude. The choice of ECFP4-based auxiliary models as nonconformity scores is specific to this study and reflects the black-box deployment scenario with VEGA models; other QSAR CP applications may equally employ ensemble variance, leverage-based AD metrics, or model-native uncertainty estimates as nonconformity scores within the same conformal prediction framework.

In both regression and classification cases, the calibration quantile *q̂* is derived exclusively from nonconformity scores computed on a held-out calibration set, not on raw predicted values or labels. Importantly, nothing is optimized to achieve coverage: *q̂* is simply the empirical quantile of the calibration scores at level ⌈(*n* + 1)­(1 – α)⌉/*n*, and coverage follows as a mathematical consequence of this rank-based construction under exchangeability. The coverage guarantee is therefore a property of the calibration procedure itself, not an optimization target and not dependent on the quality of the underlying model: a poorly performing QSAR model will produce wide intervals or large prediction sets, but the nominal coverage will still be met.

The practical workflow consisted of the following steps:

**Step 1.** **Obtain the predictions.** Use the base model for the endpoint of interest (e.g., existing VEGA model). Regression: The model predicts continuous values. Classification: The model predicts class probabilities or discrete labels (e.g., toxic vs nontoxic).

**Step 2. Nonconformity scores on the calibration set.** Compare predictions to the experimental values to quantify model error. For regression models, compute the residuals—the differences between predicted and observed values. For classification models—compute a score reflecting how unusual a predicted class is compared to the true class. These errors will form the calibration scores, which are used to determine the quantile *q*_1−α_ for the desired confidence level (e.g., 95%).

**Step 3 (optional adaptive variant). Auxiliary sigma model.** If the residual variance is not constant as a function of the predictor variable *x* (i.e., heteroscedasticity is present), then a constant-width interval is inefficient—for some inputs, the model may be very confident (small residuals), while for others, uncertainty may be high (large residuals). Train an auxiliary model (using the training set or a separate validation set if available) to predict the scale of the residuals as a function of *x* (e.g., fingerprints, descriptors, not necessarily the original model descriptors). This auxiliary model is known as “σ model”, because it estimates σ­(*x*), the expected scale (standard deviation) of residuals for input *x*, making the conformal interval adaptive. For regression, it predicts the expected residual size; for classification, it predicts the expected error or score for each chemical/class. Apply the σ model to the calibration set to obtain calibration scores (in our case, sigma-adjusted scores instead of the raw residuals).

**Step 4.** **Calibration.** From the calibration set scores (raw residuals or sigma-adjusted), compute the quantile corresponding to the desired confidence level.

**Step 5. Apply conformal prediction to new chemicals.** Compare the nonconformity score (e.g., predicted error or sigma-adjusted error) for a new chemical to the calibrated threshold. Regression: we use the approach where the prediction interval is centered on the base model prediction, with the width determined by the quantile of residuals *q*_1−α_.

$$ŷ\left(x\right)\pm q_{1-\alpha }\sigma \left(x\right)$$

Classification: the prediction set includes all classes whose predicted score is below the threshold, which is the quantile of the calibration scores corresponding to the desired confidence level (1−α).

**Summary**

• The calibration set’s nonconformity scores determine the quantile/threshold, ensuring valid coverage. The choice of the calibration set is important and should be large enough to meet the set level of coverage.

• The sigma model is optional and is used for adaptive intervals or prediction sets; it is trained on training data different from the calibration set and applied to the calibration set.

• Both regression and classification follow the same logic, with nonconformity scores calculated on the calibration set.

**QSAR Models**

We used the VEGA platform (1.2.4) as the source of QSAR models and training/test data sets. The downloadable VEGA software^33^ provides more than 100 models covering physicochemical properties, environmental fate, and toxicological endpoints. Each model is distributed with a training set, and the majority of the models also with an external test set curated by VEGA developers. Our workflow consists of four main stages: 1) training and test set extraction from VEGA software, 2) VEGA model wrapping to enable automated large-scale predictions, 3) conformal prediction calibration using external test sets using the MAPIE library,^21^ and 4) application of calibrated models. As the direct use of the VEGA GUI is impractical for large-scale evaluation, we developed a headless Java command-line wrapper for VEGA with a streaming output mode, enabling batched predictions and efficient extraction of model results, which is available at https://github.com/ideaconsult/quarkus-vega-cli. Steps 3–4 are implemented in Python, with workflow orchestration organized as a Ploomber pipeline: https://github.com/ideaconsult/qubounds.

Conformal prediction for models from an existing platform poses several challenges. First, CP requires a separate calibration data set. For each VEGA model, we split the test set provided in VEGA into calibration and test set. We assume the test set was not used for training VEGA models. The sigma models are trained using the training sets. Scores and sigma models for regression models use sigma-adjusted residuals using the standard MAPIE classes and custom sigma regression models (Random Forest, kNN, LightGBM^34^) with ECFP4 fingerprints^32^ as molecular descriptors. Since VEGA classification models output hard labels rather than probabilities for most of the models, we trained auxiliary models to predict the residuals of VEGA’s classification outputs and use these residual-based models to construct class probabilities and subsequently nonconformity scores through the LAC (Least Ambiguous Set) algorithm.^35^

The evaluation proceeded in three stages. First, auxiliary model selection is addressed by comparing candidate auxiliary models for both regression and classification tasks, using held-out interval width and generalization performance as selection criteria.

Second, the selected auxiliary models are evaluated on the VEGA training and test data sets to establish the validity and efficiency of the conformal procedure under controlled conditions where true endpoint values are available. For regression, this includes coverage and efficiency analysis across all VEGA models, characterization of data set difficulty via interval width distributions, and the relationship between interval width and the applicability domain index (ADI). For classification, the same analyses are performed using singleton rate as the efficiency metric, with coverage, set size, and singleton rate examined both globally and stratified by ADI bin.

Third, the calibrated models are applied to the large external EPA CompTox chemical inventory, where true endpoint values are considered unavailable, and evaluation is therefore restricted to efficiency and its relationship with ADI. This stage assesses whether the uncertainty structure learned on VEGA data sets generalizes to a chemically diverse regulatory inventory and provides a practically relevant demonstration of CP deployment in the absence of experimental ground truth.

### 2.1 Implementation

#### 2.2.1 Conformal Prediction for Regression Models

Three approaches to auxiliary (NCM) models were evaluated, all using ECFP4 molecular fingerprints to predict absolute residuals: RF-ECFP: Random Forest regressor, LGBM-ECFP: LightGBM regressor, and kNN-ECFP: k-Nearest Neighbors regressor (Figure 3). Each auxiliary model (σ̂) is trained to predict |y_true – y_pred| from the underlying VEGA models. Prediction intervals are constructed as [*ŷ* – *q*·σ̂, *ŷ* + *q*·σ̂], where *q* is the (1 – α)-quantile of normalized calibration conformity scores *s* = |*y* – *ŷ*|/σ̂. The adaptive behavior of the approach is illustrated in Figure 2. Data partitioning varies by data set availability: if available, an existing test set is used for calibration. If VEGA provides no test sets, the original data set is split into 80/20 (training + calibration)/test set, where training + calibration is further split into 80/20 subsets. Split conformal prediction is implemented via the MAPIE *SplitConformalRegressor* class with *ResidualNormalizedScore* at α = 0.1 (90% target coverage). Since VEGA models were already trained and produced fixed predictions, we implemented an *ExternalPredictor* wrapper that returns precomputed predictions to MAPIE while the conformal framework operates solely on the calibration/test features (ECFP4) for uncertainty estimation. This decouples the base predictor from the nonconformity model. Before training sigma models, residuals were analyzed for degeneracy (concentration near zero) via 90th/95th percentile thresholds and the fraction of exact zeros. This diagnostic informed whether epsilon regularization was necessary to prevent division-by-zero in normalized scores.

**Figure 2:**
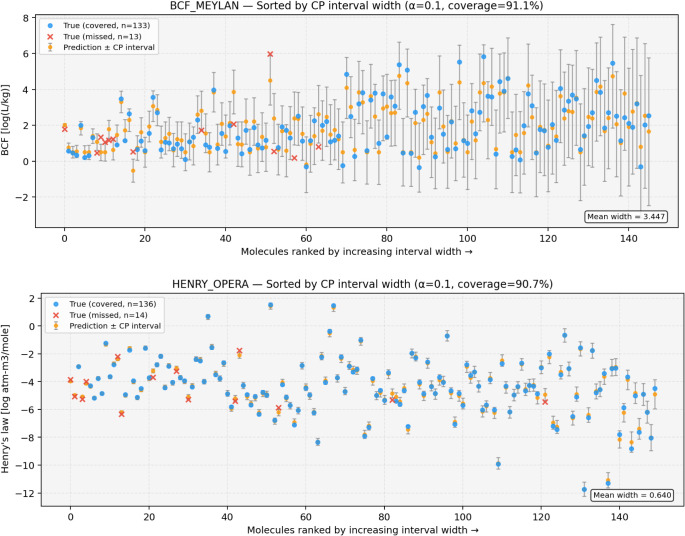
Conformal prediction intervals for two VEGA regression models illustrating the adaptive behavior of the framework. Molecules are ranked on the *x*-axis by increasing interval width; blue circles and red crosses denote covered and missed true values, respectively; orange circles are point predictions. Gray error bars are 90% CP intervals (α = 0.1). Empirical coverage meets the nominal level in both cases (BCF_MEYLAN: 91.1%, Henry’s law: 90.7%). The contrast between panels is deliberate: the wide, heterogeneous intervals of BCF_MEYLAN (mean width 3.45 log L/kg) reflect a noisy, structurally diverse endpoint, whereas the tight, nearly uniform intervals of HENRY_OPERA (mean width 0.64 log atm·m^3^/mol) reflect a smooth, well-modeled endpoint. This molecule-level variation in interval width is the result of the sigma model σ̂(*x*) trained on molecular fingerprints.

The following validation metrics are calculated: Empirical coverage—the fraction of true values within intervals; Relative interval width—the interval width normalized by the training set prediction range; and the sigma model quality—*R* ^2^, RMSE, and MAE of the residual prediction model. In addition, beyond standard coverage metrics, we provide exchangeability diagnostic metrics, implemented with Kolmogorov–Smirnov tests comparing calibration vs test conformity score distributions (*H*_0_: exchangeability). Quantile stability was assessed via Δ*q* = |q_cal – q_ref|, where q_ref is the empirical quantile on test/training sets. Large Δ*q* or significant KS *p*-values flag potential distribution shifts. In practice, auxiliary model selection involves a trade-off between generalization performance on held-out data and the resulting interval efficiency; we recommend against models with very low held-out *R* ^2^—which may approximate a global constant rather than capturing local uncertainty structure—and against highly flexible interpolators prone to overfitting, which inflate interval width despite high training *R* ^2^. The model used in the Results section is the LightGBM regressor (rlgbmecfp) as a compromise between model quality and relative interval width (Figure 3).

**Figure 3:**
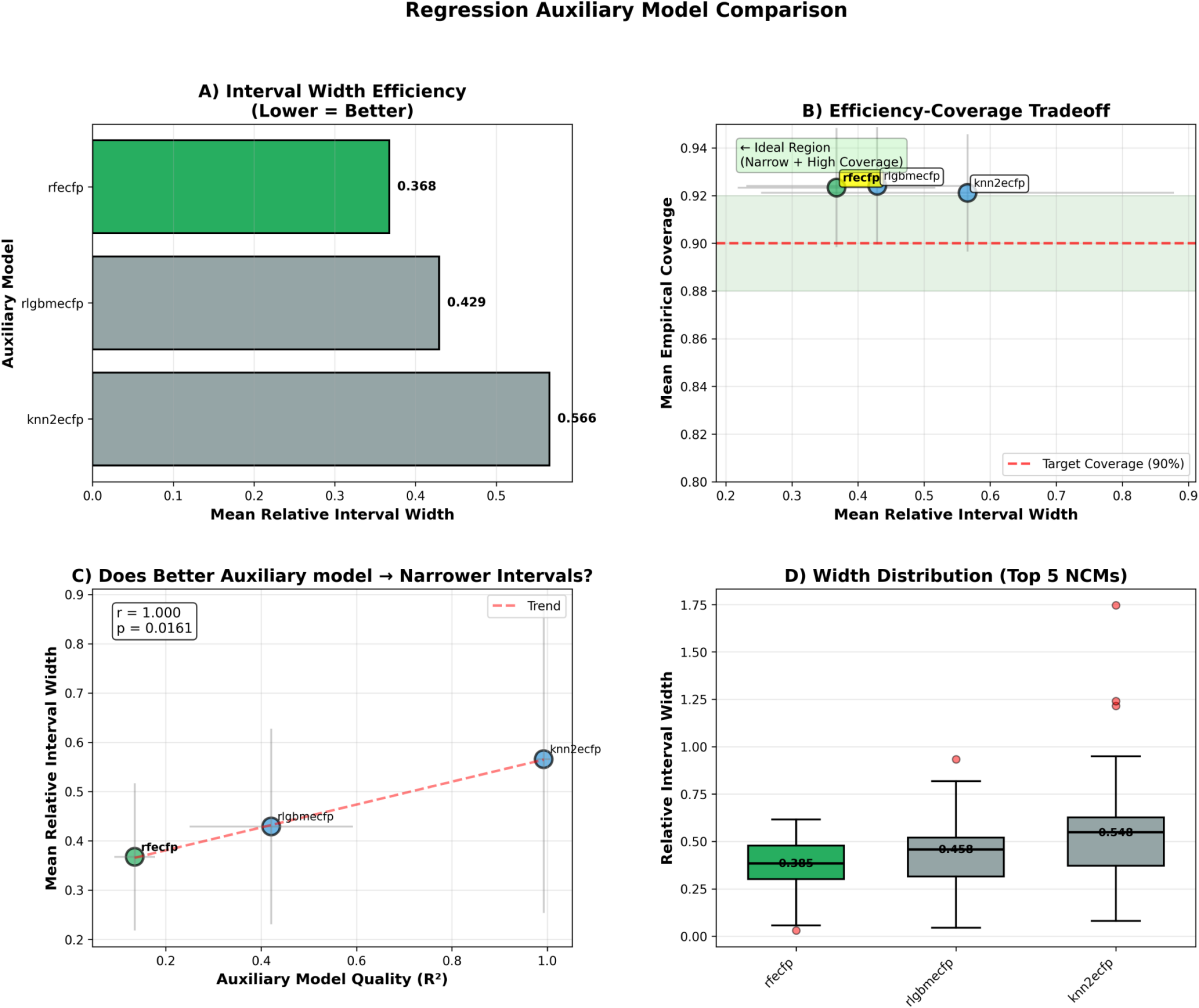
Auxiliary models comparison showing (A) interval width efficiency by auxiliary models, (B) coverage-width trade-off scatter plot with the target 90% line, (C) sigma model quality (*R* ^2^) vs interval width with correlation statistics, and (D) width distribution box plots.

We used the efficiency metric (relative interval averaged across data set) to identify the most challenging and the most well-modeled “easy” data set (Figure 4) across all auxiliary models. Overall, the different auxiliary models provide consistent results, which we verify through correlation matrices of interval widths and coverage across data sets.

**Figure 4:**
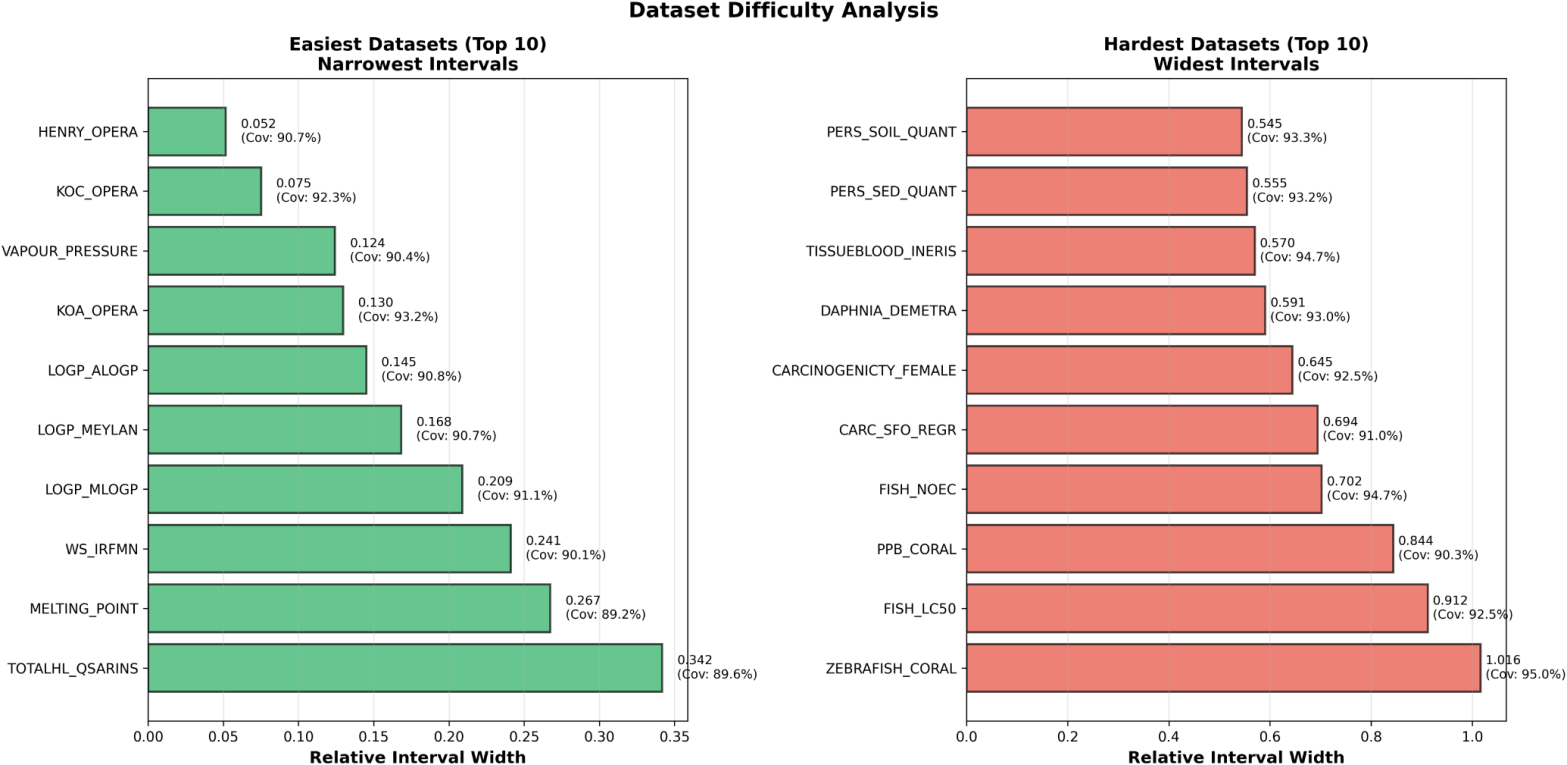
Horizontal bar charts comparing (Left) the top 10 easiest data sets with the narrowest intervals and (Right) the top 10 hardest data sets with the widest intervals, both annotated with coverage values. A relative interval of 1 means the intervals span the entire range of the response variable.

Relative interval widths averaged per data set ranged from 0.052 (easiest) to 1.016 (hardest), with a 19-fold difference between extremes. With the LightGBM-ECFP auxiliary model, all data sets but two achieved the target 90% coverage (SLUDGE_COMBASEEC50 and HYDROLYSIS_CORAL because of the small test size of 10–12 molecules). The EW_TOXICITY model is excluded because of the data set size being less than required for the target coverage level. We report detailed coverage and efficiency statistics as well as a comparison with ADI in the Results section.

All trained auxiliary models were serialized for application to external query sets, with chunked processing (10000 molecules/batch) enabling scalable deployment.

#### 2.2.2 Conformal Prediction for Classification Models

The majority of VEGA classification models output only hard class labels (e.g., “toxicity = 2”) without confidence scores or probability distributions. Standard conformal classification methods like LAC (Least Ambiguous Set) and APS (Adaptive Prediction Sets) require class probabilities (*predict_proba* in scikit-compatible outputs), making them incompatible with VEGA models. We developed a novel **Auxiliary Model-Based Pseudoprobabilistic Approach** to bridge this gap. The core innovation converts hard predictions into synthetic probability distributions using a two-stage approach with an auxiliary sigma model of either regression or classification type. This approach benefits from the ordinal structure of typical toxicity classifications, where the severity of prediction errors matters (predicting class 1 when true is class 3 is worse than predicting class 2).

Stage 1: Ordinal Distance Modeling. Auxiliary models predict the ordinal distance between predicted and true classes: Classifier-based sigma models directly output discrete probability distributions via multiclass classification: σ­(ECFP) → *P*(distance = *k*) where *k* ∈ {0,1,2,3}. Regressor-based σ-models predict continuous expected distance σ­(ECFP) – *d̂*, then convert to probabilities via Gaussian-like decay: *P*(distance = *k*) ∝ exp­(−|*k* – *d̂*|), followed by normalization.

Stage 2: Distance Probabilities to Class Probabilities. For a molecule with predicted class *ŷ* and distance probabilities *P*(distance = *k*) from the σ-model, we map to class probabilities: *P*(class = *j* | molecule, *ŷ*) = *P*(distance = |*j* – *ŷ*| | molecule).

Example for a classifier-based model: If *ŷ* = 1 and the σ-model outputs [*P*(*d* = 0) = 0.6, *P*(*d* = 1) = 0.3, *P*(*d* = 2) = 0.08, *P*(*d* = 3) = 0.02], then

• *P*(class = 0) = *P*(distance = |0–1| = 1) = 0.3

• *P*(class = 1) = *P*(distance = |1–1| = 0) = 0.6 # the highest (predicted class)

• *P*(class = 2) = *P*(distance = |2–1| = 1) = 0.3

• *P*(class = 3) = *P*(distance = |3–1| = 2) = 0.08

After normalization (sum = 1.28): *P* = [0.234, 0.469, 0.234, 0.063].

These pseudoprobabilities encode both the hard prediction (highest at *ŷ*) and σ-model uncertainty. Classes equidistant from the predicted class *ŷ* receive equal probability, respecting ordinal symmetry.

Two auxiliary model paradigms are evaluated for predicting ordinal distances, all using ECFP4 fingerprints: classifier-based auxiliary models (multiclass classification of discrete distances, using scikit-learn Random Forest classifier, Gradient Boosting, kNN) and regressor-based auxiliary models (continuous distance estimation using RF, GB, kNN). Classifier models are trained on integer distance labels (0, 1, 2, 3) and output discrete probability distributions via “predict_proba­()”. Regression models predict continuous distance values via “predict­()”. To obtain pseudoprobabilities, we apply Gaussian-like decay around the predicted distance: *P*(distance = *k*) ∝ exp­(−|*k* – *d̂*|), then normalize. A separate implementation path handles models where probability outputs are available, using standard LAC conformity scores^35^ based on class probabilities without auxiliary model training.

MAPIE conformal framework integration. A wrapper class *NCMProbabilisticClassifier* implementing “predict_proba­()” was created to interface the auxiliary model with MAPIE: 1. Retrieve hard prediction *ŷ* from a file with precomputed predictions; 2. Compute sigma model distance probabilities from ECFP4; 3. Convert to class probabilities via distance mapping; 4. Return a synthetic probability vector. The wrapper enables MAPIE *SplitConformalClassifier* to use the standard LAC conformity score, calibrate the threshold τ on the calibration set, and produce prediction sets {*j*: score­(*j*) ≤ τ}.

The calibration strategy is identical to the regression approach—the test set is used for calibration, and the target coverage is 90% (α = 0.1). The following validation metrics are calculated: Empirical coverage—the fraction of true classes within the prediction set; Average set size—the mean number of classes per prediction set (an efficiency metric); Point accuracy—classification accuracy (singleton sets only); Off-by-one accuracy—the fraction where |y_true – *ŷ*| ≤ 1; Mean ordinal distance—Average |y_true – *ŷ*| across the test set; Singleton efficiency or Singleton rate—the fraction of correct predictions with single-class sets; Auxiliary model quality = *R* ^2^, RMSE, MAE of ordinal distance predictions.

Classifier-based auxiliary models, in general, outperformed regressor-based models. The ability to predict probability distributions over discrete ordinal distances is likely superior to point estimates of the expected distance. Similar to the regression case, the selection of the auxiliary model is a trade-off between efficiency and coverage (Figure 5), and the gradient boosting classifier (**cgbecfp**) is selected, which is used in the Results section.

**Figure 5:**
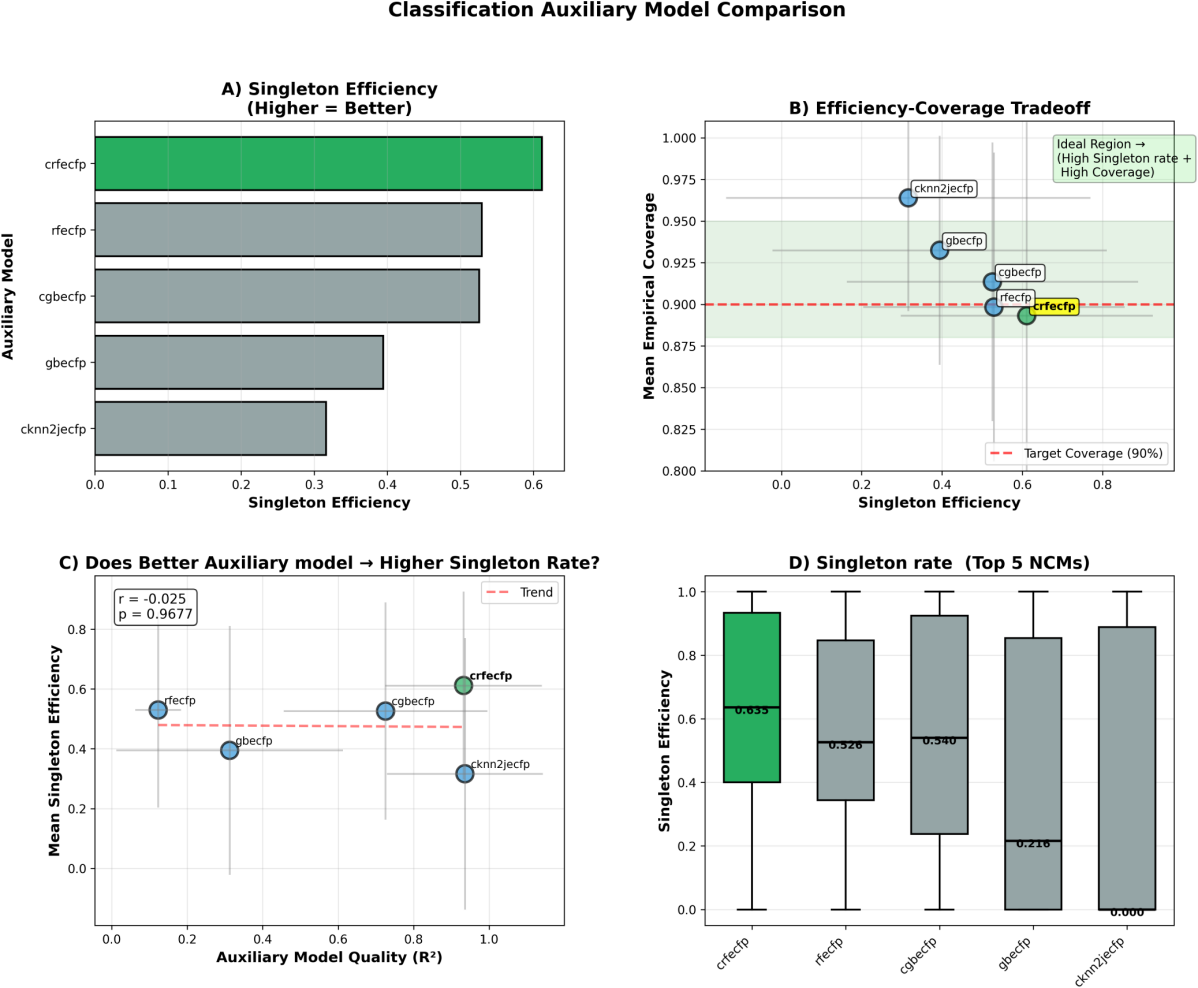
Comparison of auxiliary models showing (A) singleton efficiency by auxiliary models, (B) coverage-singleton trade-off scatter plot with the target 90% line, (C) sigma model quality (*R* ^2^) vs singleton rate with correlation statistics, and (D) singleton rate distribution box plots.

## 3 Results

### 3.1 Regression Models Findings on VEGA Training and Test Sets

We provide a summary of the validity (coverage) and efficiency (interval width) of conformal prediction applied across all VEGA regression QSAR models (Figure 6), using the selected LightGBM auxiliary model and combined training and test sets. Panel A shows empirical coverage for each data set, with a dashed horizontal line indicating the nominal 90% target. All models except one fall within the 85–95% range, demonstrating that conformal prediction consistently achieves near-nominal coverage across chemically and biologically heterogeneous data sets.

**Figure 6:**
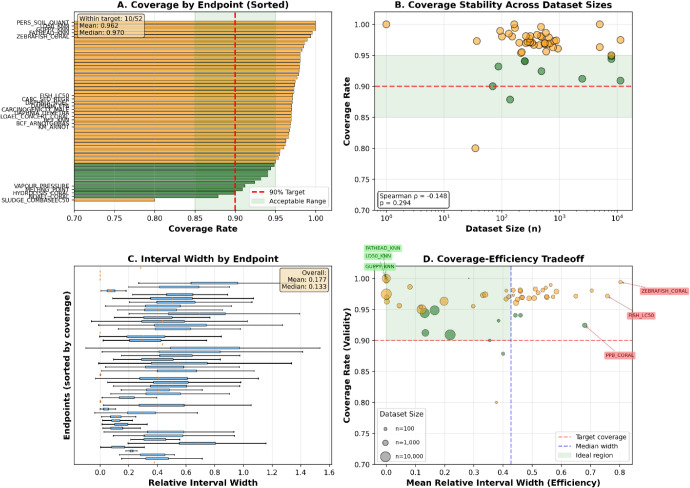
Coverage and efficiency analysis of VEGA regression models. Panel A shows that most data sets achieve empirical coverage close to the 90% target, indicating consistent reliability. Panel B demonstrates that coverage remains stable across a wide range of data set sizes. Panel C shows that mean interval widths vary by endpoint, reflecting intrinsic differences in predictability and noise. Panel D illustrates the coverage–efficiency trade-off, with most data sets exhibiting both good coverage and reasonably tight intervals.

The variation in coverage across ADI ranges (88.8% for low ADI to 99.2% for high ADI) is small compared to the variation in ADI itself (spanning from 0 to 1) (Figure 7). This demonstrates the robustness of conformal prediction: even when ADI suggests predictions are outside the reliable domain, conformal intervals still provide near-nominal coverage. The slight undercoverage at low ADI (88.8% vs 90% nominal) and overcoverage at high ADI (99.2%) reflect the conservative nature of the conformal calibration. Importantly, all ADI ranges achieve nominal coverage providing users with reliable uncertainty estimates regardless of applicability domain estimation. Instead of declaring predictions unreliable (low ADI), the conformal prediction statement is “These predictions are uncertain (wide intervals), here’s the quantified uncertainty—and they still achieve 88.8% coverage”.

**Figure 7:**
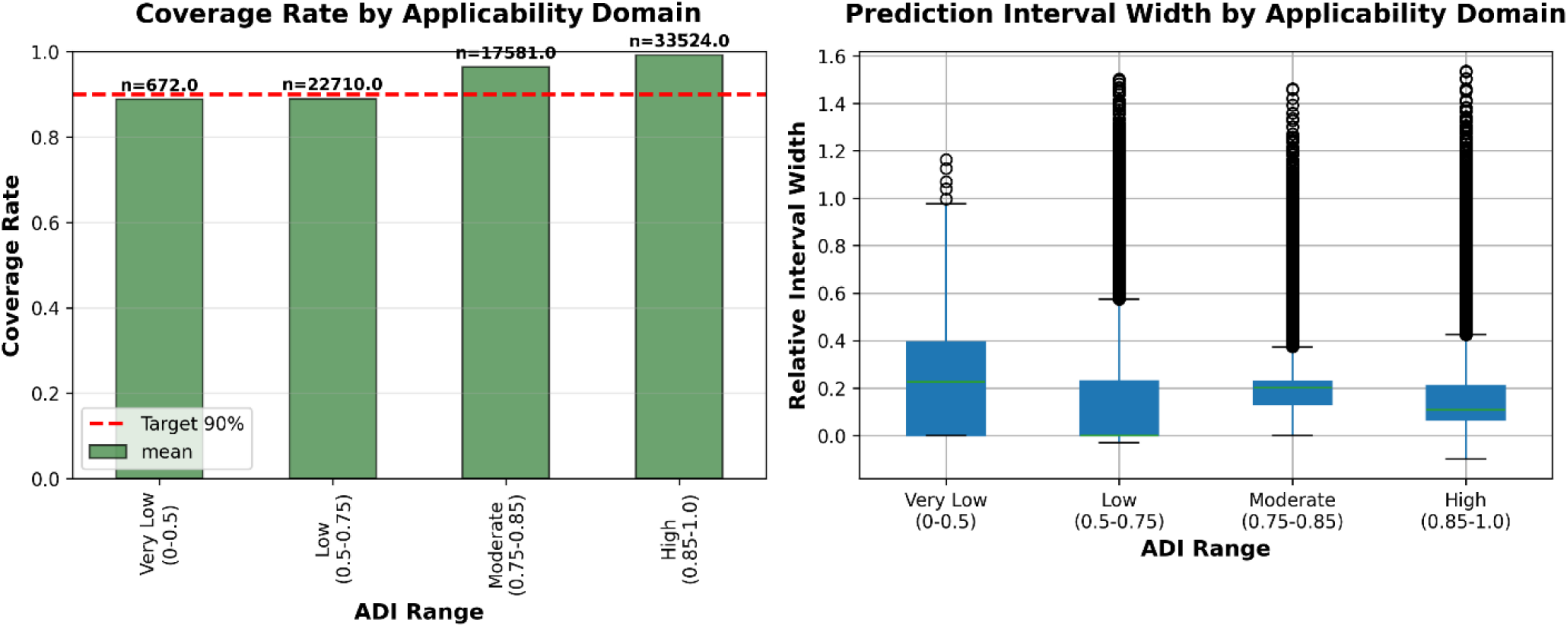
Coverage rate and prediction interval width for VEGA regression models (all training and test sets combined) stratified by the applicability domain index (ADI). (Left) Fraction of true values falling within prediction intervals for each ADI range. The red dashed line indicates the target 90% coverage level. (Right) Distribution of prediction interval widths across ADI ranges. Sample sizes (*n*) are shown above each bar. Higher ADI values indicate predictions closer to the model’s training domain.

Conformal prediction intervals naturally adapt to domain similarity without requiring explicit thresholds or AD metrics (Figure 8). Across 52 QSAR endpoints, 80% show a negative correlation between ADI and interval width (median Spearman rank ρ = −0.327), with 58% highly significant (*p* < 0.001). This demonstrates that conformal prediction automatically provides wider intervals for predictions flagged as unreliable and narrower intervals for predictions flagged as reliable (high ADI). Of note, ADI is an independent metric provided by VEGA; it was not used in the calibration process at all. The correlation strength varied substantially across endpoints (ρ = −0.7 to +0.06), with the strongest negative correlations observed for physicochemical properties like vapor pressure and logP predictions. Data set size influenced correlation strength: larger data sets showed stronger ADI-interval relationships (meta-correlation ρ = −0.43), with endpoints containing >1000 samples predominantly showing robust negative correlations. Small data sets (*n* < 100) show high variability, including occasional positive correlations, likely reflecting insufficient chemical space coverage. Interestingly, the MELTING model show a moderate negative correlation (ρ ≈ −0.2), despite having the largest combined training/test data set. A possible explanation, corresponding to the known difficulty of modeling the melting point, is the potential heterogeneous uncertainty sources beyond structural similarity.

**Figure 8:**
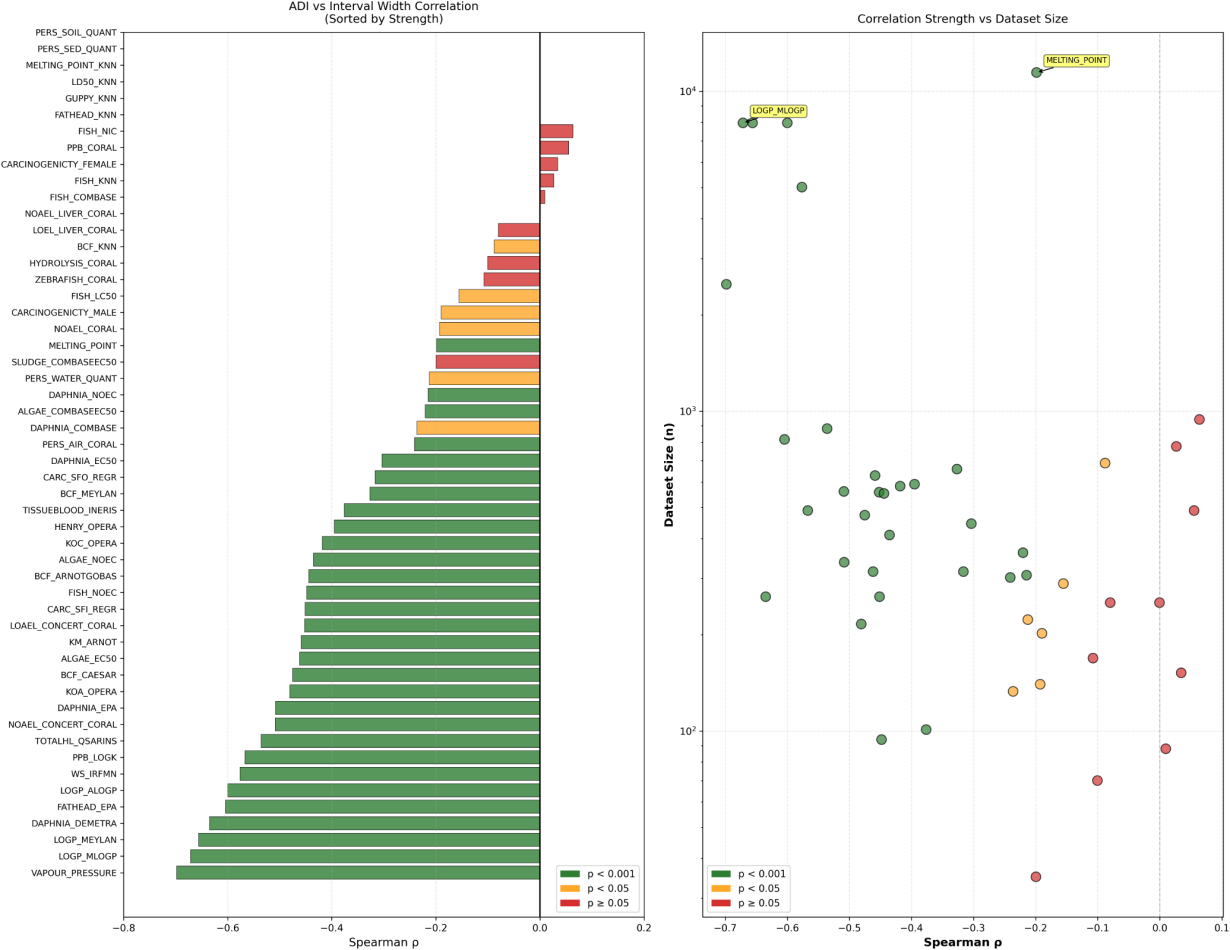
Correlation between applicability domain index (ADI) and conformal prediction interval width across 52 VEGA QSAR regression models. (Left) Spearman’s rank correlation coefficients (ρ) sorted by strength, with color indicating statistical significance (green: *p* < 0.001, orange: *p* < 0.05, red: *p* ≥ 0.05). (Right) Relationship between correlation strength and data set size (*n*).

Overall, these results demonstrate that conformal prediction intervals naturally adapt their width in response to domain similarity, providing wider intervals for uncertain predictions without requiring an explicit domain index and thresholds.

### 3.2 Regression Models Findings: Results on EPA CompTox Data Set

We evaluated conformal prediction uncertainty quantification across 44 QSPR/QSAR regression models and ∼500 K chemicals from the EPA CompTox data set, generating 22 million predictions. The data sets are available on Zenodo (v2).^36^ Prediction intervals were normalized by the training set range (max–min) to enable cross-model comparison. The analysis demonstrates that CP provides robust uncertainty estimates that strongly reflect the model applicability domain. The key findings are described below and in Figure 9 and Figure 10.

**Figure 9:**
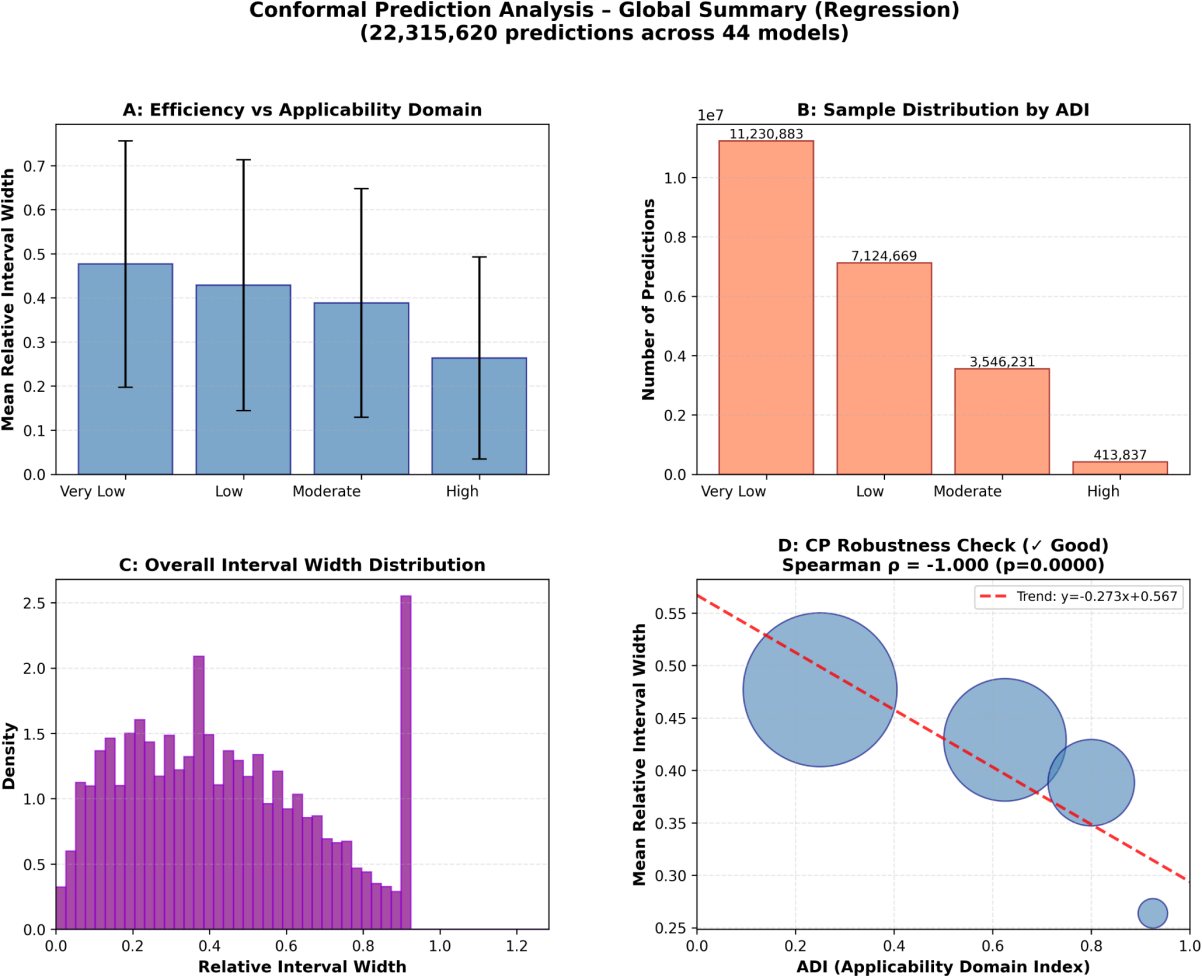
Global summary of conformal prediction uncertainty for regression models across applicability domains for EPA CompTox chemicals. Bar plots show mean uncertainty (relative interval width for regression) with standard deviation error bars (panel A) and sample counts by ADI bin (panel B). The histogram (panel C) displays the approximate global distribution of the relative interval width. A scatter plot with a trend line (panel D) illustrates the relationship between mean relative interval width and ADI bin center, with Spearman rank correlation assessing the robustness of conformal prediction in relation to the applicability domain index.

**Figure 10:**
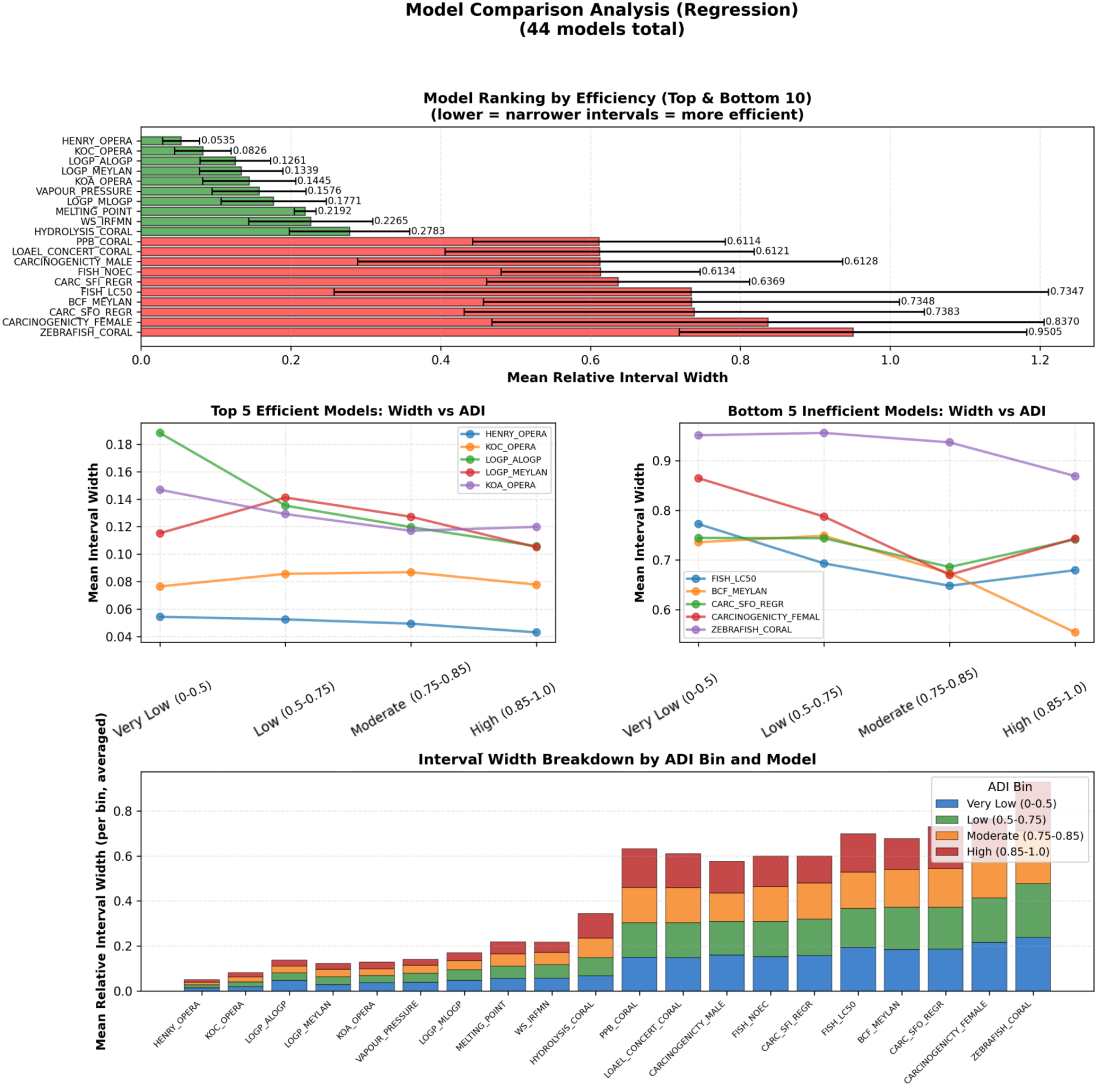
Model comparison of conformal prediction efficiency (relative interval width) for regression models applied to EPA CompTox chemicals. A horizontal bar chart ranks models by overall relative interval width with standard deviation. Line plots compare relative interval width vs ADI for the five easiest and five most difficult models (middle panels). A bar chart (bottom panel) depicts approximate relative interval width across ADI bins for the 10 top-performing and 10 worst-performing models.

Excellent CP Robustness: the correlations are estimated from averaged quantities in ADI bins. Relative interval width shows a perfect negative rank correlation with the applicability domain index (Spearman ρ = −1.00, *p* < 0.001), confirming that CP uncertainty estimates reliably identify when models are operating within their applicability domain. The strong linear correlation demonstrates this relationship holds across the full ADI spectrum. ADI bins follow the VEGA definition,^7^ where High is ADI > 0.85, Moderate is ADI in [0.75, 0.85], and Low ADI is elsewhere. To facilitate analysis and visualization, the low range was split into Low [0.5–0.75] and Very Low ADI under 0.5.

Applicability Domain Coverage: Over half of the predictions (50.3%) fall in the Very Low ADI category [0–0.5] with a mean relative interval width of 0.47 ± 0.27 (47% of the training range). As applicability increases through Low (31.9%), Moderate (15.9%), and High (1.8%) ADI categories, the mean relative interval width decreases progressively (0.42 to 0.38 to 0.26), demonstrating that CP appropriately assigns narrower prediction intervals to chemicals within the model’s domain of applicability. Note that these are global statistics across all models and all compounds, though model-specific statistics are similar.

Model-Specific Performance Heterogeneity (Figure 10): Prediction certainty varies substantially across physicochemical properties and toxicity endpoints. Physicochemical property models exhibit the highest certainty: Henry’s law constant (HENRY_OPERA, mean width = 5.4% of range), soil sorption coefficient (KOC_OPERA, 8.3%), and partition coefficients (LOGP models, 13–17%). Environmental fate parameters show moderate uncertainty (hydrolysis, persistence: 28–28% of range), while acute aquatic toxicity models vary widely (fish: 34–73%, daphnia: 43–56%, algae: 40–57%). Chronic toxicity endpoints (NOEC/LOEC) and complex biological endpoints (carcinogenicity, developmental toxicity) exhibit the highest uncertainty (57–84% of range). kNN regression models are excluded from this figure, as in most VEGA kNN models, all errors are close to zero in the training set and it is not possible to derive reliably an auxiliary model and compute conformal intervals. In such cases, cross-conformal prediction is recommended, but it would mean retraining the model, which is not in-scope of this work.

Practical Implications for Risk Assessment: Only 1.8% of predictions achieve High ADI status (mean interval width = 26% of training range), indicating that most QSPR predictions for the external data set involve significant extrapolation. The majority of predictions (51%) in Very Low ADI with intervals spanning ∼47% of the training range require careful interpretation. These results emphasize the importance of uncertainty quantification for regulatory applications, where CP intervals can inform testing priorities and confidence levels in predictive assessments instead of disgarding the prediction due to “out of applicability domain” flags.

Physicochemical vs Biological Property Prediction: The clear performance gradient from physicochemical properties (5–22% interval width) to environmental fate (28–42%) and then to acute toxicity (34–56%) and finally to chronic toxicity/carcinogenicity (57–84%) reflects fundamental differences in endpoint predictability, data quality, modeling challenges, and inherent biological variability. This hierarchy provides guidance for model selection and data-gap-filling strategies.

### 3.3 Classification Findings: Training and Test Sets

To analyze efficiency, we report efficiency diagnostics (Figure 11 bottom) based on the singleton rate, defined as the proportion of predictions for which the conformal prediction set contains exactly one class label. A singleton prediction represents the highest possible efficiency: the model commits to a single class with the required coverage guarantee. A typical efficiency metric is mean label set size; however, LAC may produce empty label sets; therefore, the singleton rate provides a better measure of how often the model is taking a confident decision.

**Figure 11:**
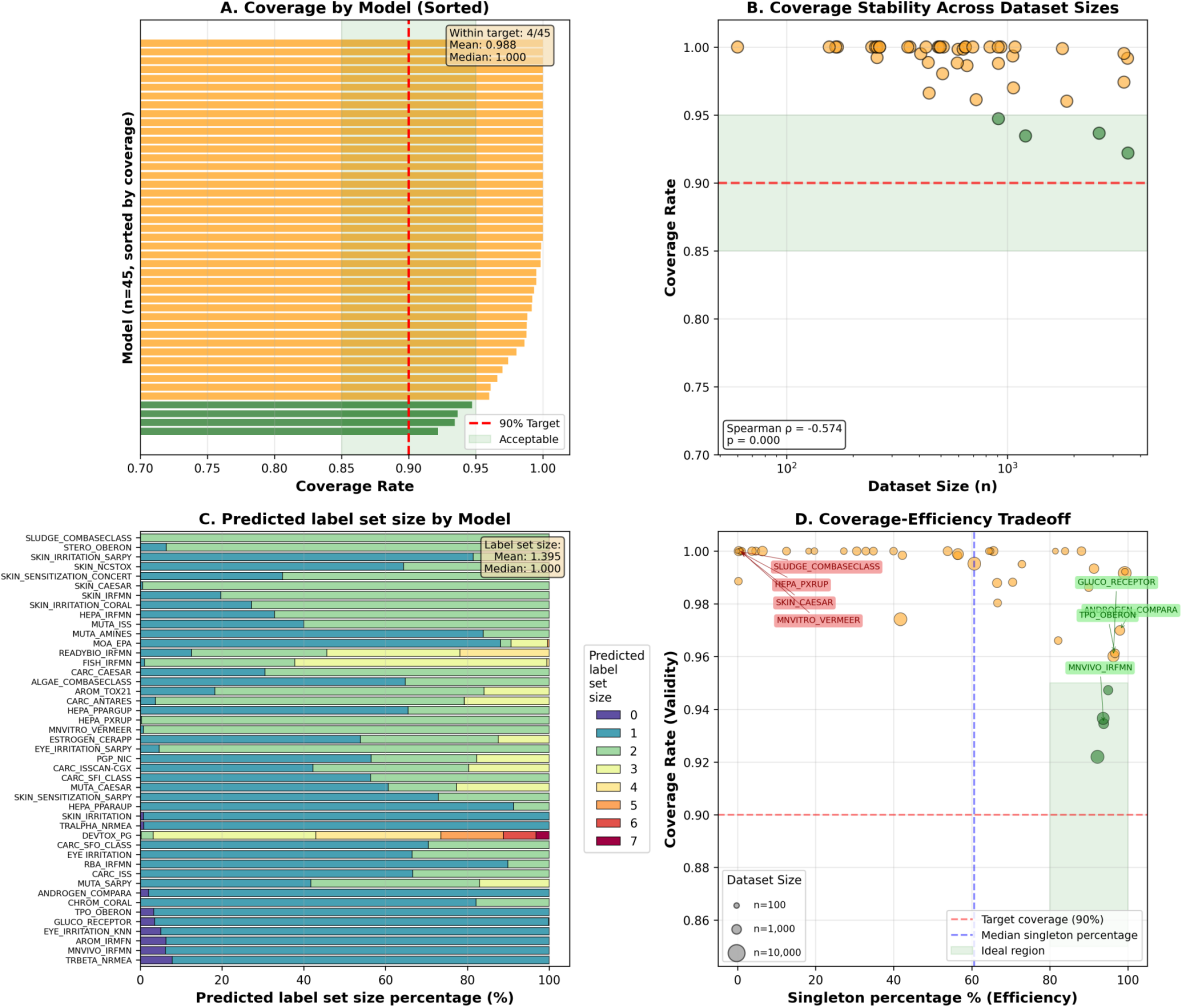
Coverage and efficiency analysis of VEGA classification models. Panel A shows that all data sets achieve empirical coverage at and above the 90% target. Panel B demonstrates that coverage remains stable across a wide range of data set sizes. Panel C shows the distribution of prediction label set sizes across endpoints. Panel D illustrates the trade-off between coverage and efficiency (singleton predictions). Bubble size reflects data set size.

A larger prediction set (containing multiple class labels) indicates that the model cannot confidently discriminate among those classes with the required coverage guarantee, whereas a singleton set, containing exactly one label, indicates that the model is decisive: it can exclude all other classes while still maintaining the coverage. A prediction set of size zero, which can arise under LAC when the nonconformity scores are unusually low, indicates that even the empty set satisfies the coverage threshold and indicates an atypical compound.

High coverage rates (Figure 11, Figure 12) likely reflect both (1) overly confident pseudoprobabilities used in the LAC framework and (2) exchangeability violations in the predefined VEGA splits. The auxiliary models, trained to predict ordinal distances, may produce concentrated probability distributions that translate to high-conformity scores in the LAC framework, resulting in conservative prediction sets. Further investigations to retrain the models with random splits and cross-conformal predictions might stabilize the results.

**Figure 12:**
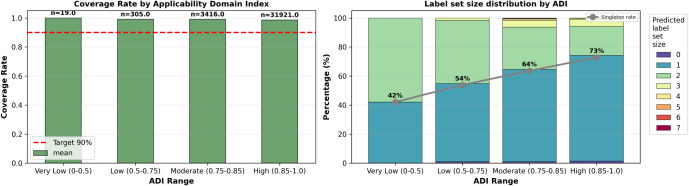
Coverage and efficiency analysis of the VEGA classification models across Applicability Domain ranges. Empirical coverage rate stratified by ADI bins, with the nominal target level (90%) indicated by the dashed line. Coverage remains stable across the domain, indicating valid calibration of the conformal predictors. The distribution of prediction set sizes across ADI bins, expressed as percentages. Singleton predictions (set size = 1), corresponding to confident classifications, increase with ADI. The overlaid line shows the singleton rate, highlighting a clear monotonic trend from low to high ADI (42–73%).

We compute the singleton rate per data set and examine its monotonic association with the applicability domain index (ADI) using Spearman rank correlation (Figure 13). A positive ρ indicates that compounds with higher ADI—closer to the training distribution—tend to receive singleton predictions more often, while a near-zero or negative ρ suggests that prediction certainty does not associate with the applicability domain index. Similarly to regression, there is a strong correlation of the classification efficiency metric to applicability domain indices.

**Figure 13:**
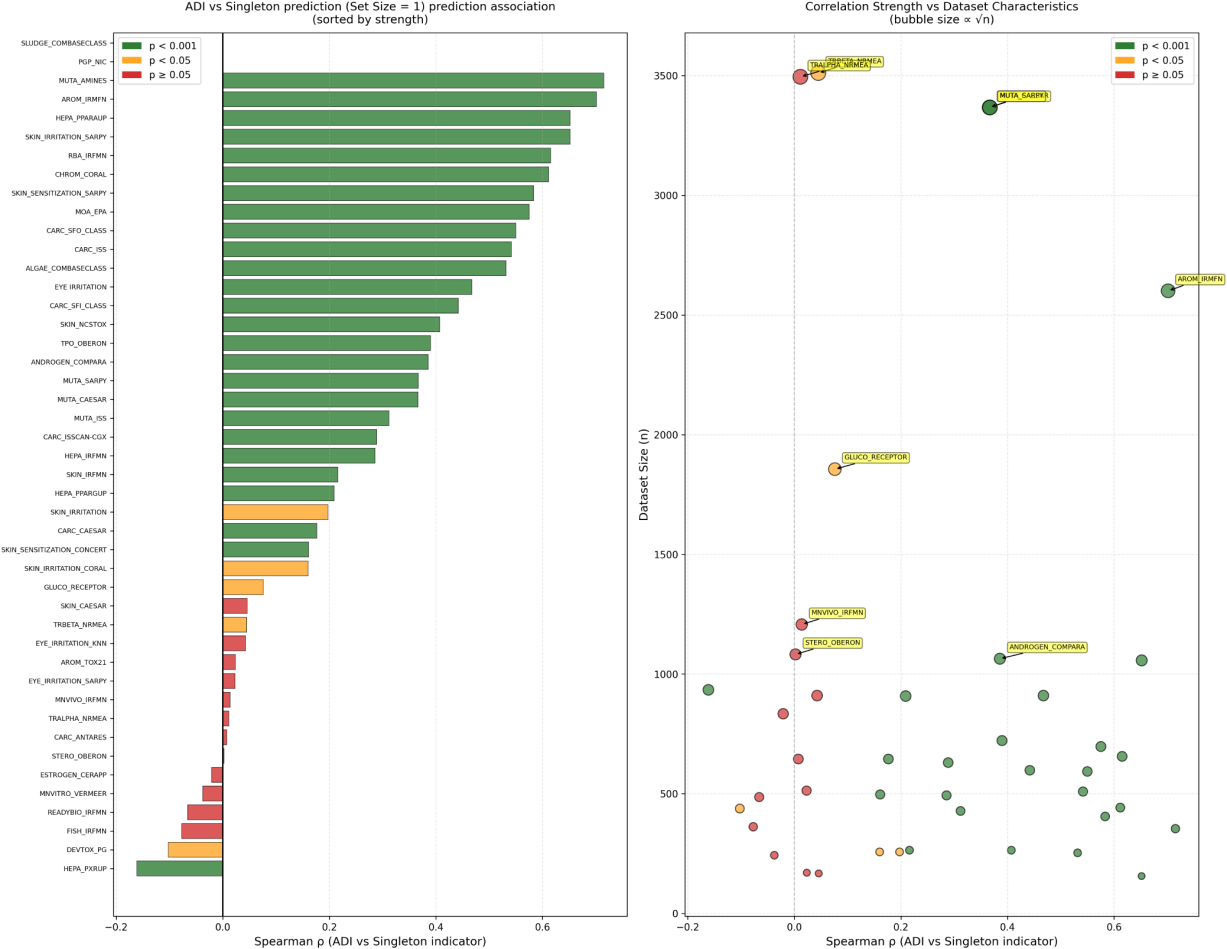
Association between applicability domain index (ADI) and efficiency (singleton predictions) across 45 QSAR classification models. (Left) Spearman correlation coefficients (ρ) between ADI and the occurrence of singletons (binary indicator), sorted by strength and color-coded by significance (green: *p* < 0.001; orange: *p* < 0.05; red: *p* ≥ 0.05). The vast majority of models exhibit a positive association, indicating that singleton predictions are more frequent in high ADI regions—Median ρ: 0.216, Range: [−0.067, 0.721]. (Right) Relationship between data set size and association strength ρ. Overall, these results demonstrate that conformal prediction sets naturally adapt their width in response to domain similarity.

### 3.4 Classification Findings with EPA CompTox Data Sets

To evaluate the real-world applicability, we applied the framework to 500 K compounds from EPA CompTox with 42 toxicity classification models resulting in over 20 million predictions. The data sets are available on Zenodo.^36^ The analysis demonstrates that CP provides robust uncertainty quantification that properly reflects the applicability domain of the QSAR models. The key findings are described below and illustrated in Figure 14.

**Figure 14:**
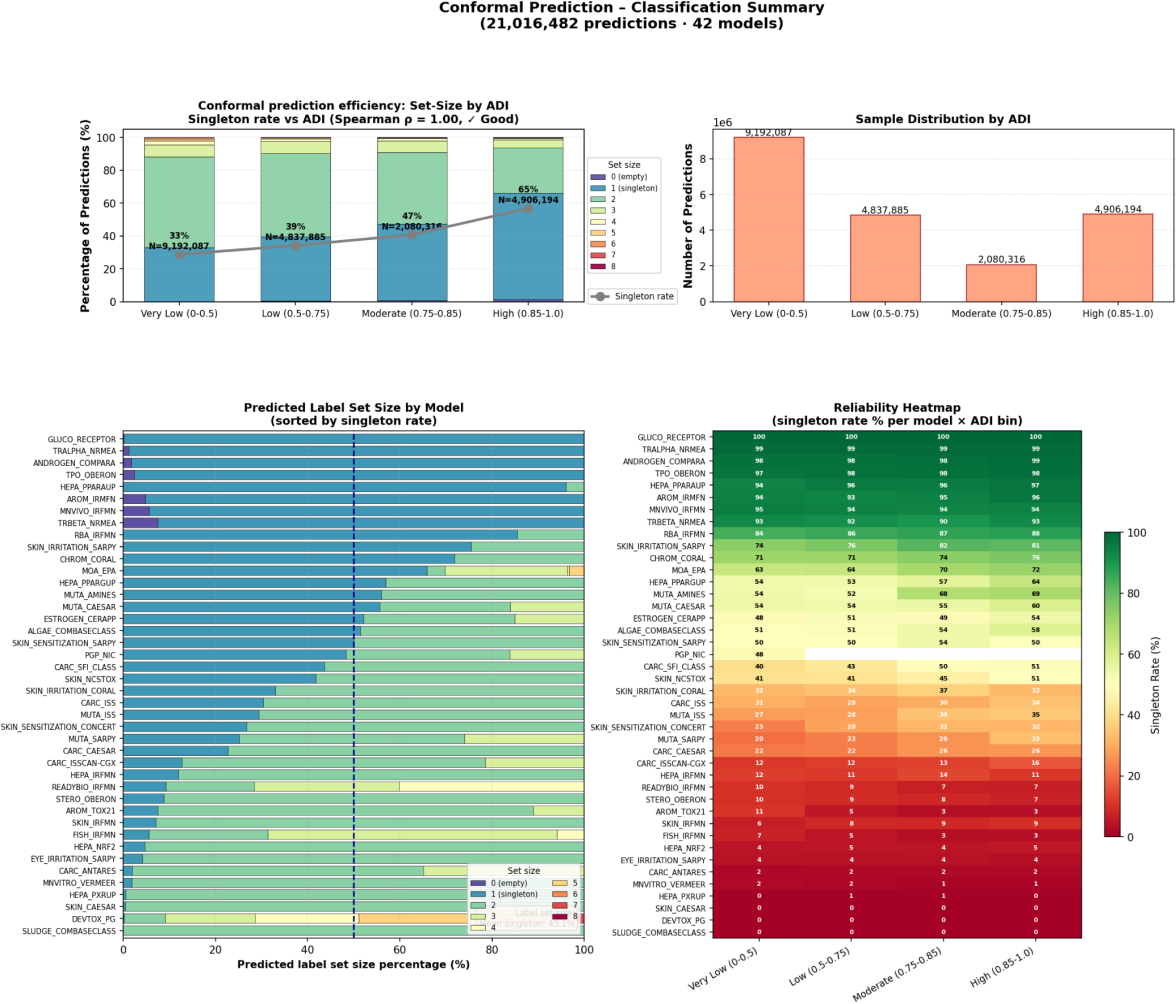
Conformal prediction efficiency summary for classification models. Top left: Distribution of prediction set sizes stratified by applicability domain index (ADI) bin, with singleton rate (percentage of unambiguous single-class predictions) overlaid. The singleton rate increases monotonically with ADI (Spearman ρ = 1.00), from 33% for compounds with very low ADI to 65% for compounds with high ADI. Top right: Sample size per ADI bin. Bottom left: Per-model distribution of prediction set sizes, sorted by singleton rate, revealing large differences in model confidence across endpoints. Bottom right: Reliability heatmap of singleton rate per model and ADI bin.

Application to the EPA CompTox inventory (which we treat as an external data set without experimental values, therefore, empirical coverage cannot be assessed) demonstrates the framework in its most practically relevant deployment scenario. In the absence of ground truth, prediction set size serves as the sole uncertainty metric: a singleton prediction indicates the model assigns the compound unambiguously to one class at 90% confidence, while multiclass or empty sets flag compounds requiring further inspection. The monotonic increase in the singleton rate with ADI across 21 million predictions confirms at scale what was established on the VEGA test sets: conformal efficiency faithfully reflects structural proximity to the training domain. Critically, this relationship emerges without any optimization but as a consequence of using ECFP-based auxiliary models as nonconformity scores, which encode the same structural similarity intuition underlying AD assessment. The evaluation represents a challenging (but typical) scenario: 43% of predictions had very low ADI (<0.5), reflecting chemicals dissimilar to the training data.

For these compounds, multiclass prediction sets are the expected and appropriate output. Unlike a binary AD flag that discards a prediction entirely when a compound falls outside the defined domain, a multiclass prediction set retains and communicates the available information—indicating which classes remain plausible at the requested confidence—allowing the end user to make an informed decision rather than receiving no output at all. Model-specific performance is illustrated in Figure 14 bottom; receptor binding models (GLUCO_RECEPTOR and TRALPHA_NRMEA, etc.) maintain high singleton rates even at low ADI, suggesting robust generalization; on the opposite scale, models such as SLUDGE_COMBASECLASS have zero singletons across all ADI bins, indicating these low-confidence models. This heterogeneity likely reflects differences in training data quality, coverage, and endpoint complexity.

The Developmental/Reproductive Toxicity library PG (DEVTOX_PG) model is a special case, as it is an expert-defined decision tree^37^ classifying chemicals into 25 categories based on structural rules. While it is already impressive that the pseudoprobabilities derived from the novel ordinal distances and conformal prediction can handle such cases, the ordinal distance concept may not be optimal for structural categories. The mutagenicity (Ames test) model (SarPy-IRFMN) (MUTA-SARPY) is another such case, composed of 112 structural rules.^38^

## 4 Discussion

### 4.1 Interpretation of Conformal Prediction Outputs

Conformal prediction intervals and prediction sets communicate uncertainty in an immediately actionable form rather than as global-model-level statistics. We provide illustrative examples to demonstrate how such set-valued predictions can be interpreted in decision-making. For regression, the interval width relative to the training data range is a practical guide to reliability.

The NOAEL_LIVER_CORAL model predicts Liver NOAEL [log (mg/kg bw)] = 2.0935 with ADI = 1 for Trifluralin (DTXSID4021395) (Figure 15 left) with a 90% conformal interval [0.89, 3.30], spanning more than 70% of the training data range [−0.43, 3.5]. The experimental value is reported as 2.188, which is covered by the interval. Despite an ADI of 1.0, this interval indicates that the model’s uncertainty for this compound is too large to support a meaningful conclusion. Similarly, the VEGA MELTING_POINT model has a 0.22 average relative interval width of 90% certainty, which translates to 159 °C in absolute terms—this is uncertainty that makes no physical sense. Thus, the CP framework acts as a transparency filter, quantifying when a model’s knowledge of a specific chemical space is too sparse to support meaningful conclusions.

**Figure 15:**
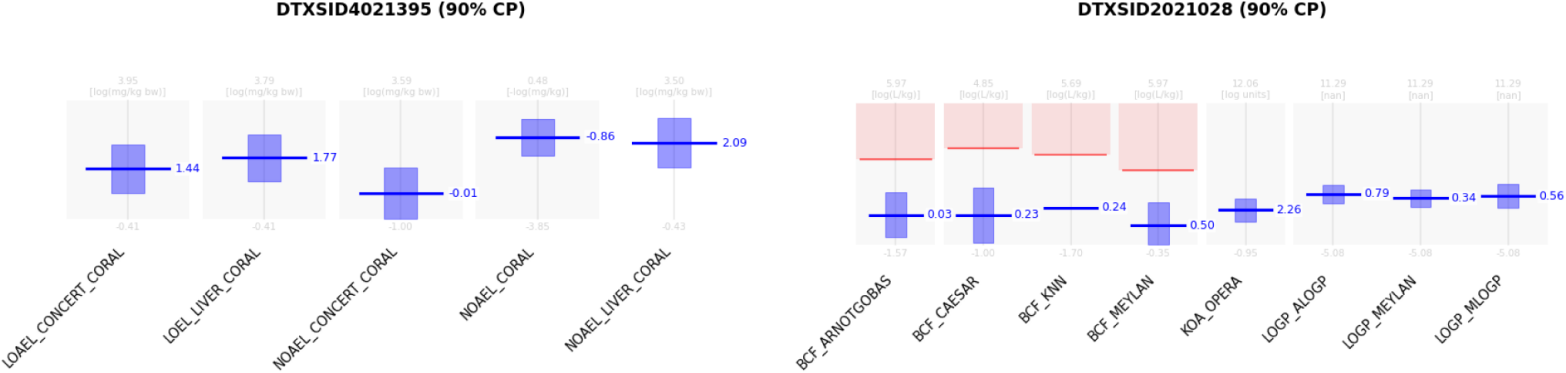
Model predictions with 90% conformal prediction (CP) intervals for two chemicals across multiple endpoints. Left panel: DTXSID2021028. Right panel: DTXSID4021395. For each endpoint, the central marker denotes the point prediction, while the shaded bars represent the associated 90% CP interval, reflecting uncertainty calibrated via conformal prediction. Endpoints include bioconcentration factors (BCF), bioaccumulation-related properties, physicochemical properties, and oral toxicity dose levels (LOAEL/NOAEL) where applicable. Values shown next to markers indicate predicted magnitudes on the corresponding model scale. For BCF, the threshold of 3 is indicated.

For borderline cases, the interval position relative to a regulatory threshold is often more informative than the width alone (Figure 15 right). For BCF, a threshold of 3 (log units) separates bioaccumulative from nonbioaccumulative substances; an interval lying entirely below the threshold supports a negative conclusion, one crossing it warrants further testing, and one entirely above it supports a positive conclusion regardless of the exact value. This threshold-referenced interpretation requires no additional statistical judgment and is directly applicable to batch screening workflows.

Experimental uncertainty provides an important external reference for interpreting conformal prediction intervals. For a 90% CP interval, about 10% of future observations are expected to fall outside. For example, the HENRY_OPERA model predicts log *H* = −4.94 [log atm·m^3^/mol] for *N*-Nitrosodiethylamine (DTXSID2021028) with ADI = 0.4 (low reliability). The 90% conformal interval is estimated as [−5.39, −4.49] log atm·m^3^/mol. The experimental value (−5.44) is only 0.05 log units outside this range, which is within typical experimental uncertainty, indicating borderline but acceptable agreement, not necessarily model failure. Standard conformal prediction does not explicitly separate experimental and model uncertainty components, as prediction intervals are derived empirically from calibration residuals. Consequently, experimental variability is implicitly reflected in the residual distribution used for calibration. In principle, assay-specific uncertainty estimates could be incorporated as normalization factors in the nonconformity score, but such estimates are generally unavailable for the data sets used here and are left as a direction for future work.

### 4.2 Classification Prediction Sets

For classification endpoints, prediction sets communicate uncertainty beyond a binary correct/incorrect call. For 2,3,4-trichloroaniline evaluated by the FISH_IRFMN acute fish toxicity model (LC50 classes: Toxic-1 < 1 mg/L, Toxic-2 1–10 mg/L, Toxic-3 10–100 mg/L, NON-Toxic >100 mg/L) with ADI = 0 (out of domain), the model predicts class Toxic-1, while the true class is Toxic-2. The 90% conformal prediction set is {Toxic-1, Toxic-2}: the point prediction is wrong, yet the set contains the true class—the coverage guarantee working as intended. Crucially, the two-class set retains regulatory meaning: both included classes fall within the acute toxicity range (LC50 < 10 mg/L), so the prediction set supports a conservative hazard conclusion regardless of which class is true. A point prediction alone would have misclassified the compound; the prediction set preserves the correct regulatory interpretation. Unlike a binary AD flag that would discard this prediction entirely at ADI = 0, the prediction set retains and communicates all available information, indicating which classes remain plausible at the requested confidence and allowing the end user to make an informed decision rather than receiving no output.

### 4.3 Conformal Prediction and Applicability Domain

Applicability domain assessment was designed to quantify prediction reliability, yet in practice it yields heuristic composite scores with arbitrary thresholds that provide no formal statistical guarantee. The strong correlations observed between conformal efficiency metrics and VEGA’s ADI—both for regression interval width and classification singleton rate—confirm that both approaches respond to the same underlying drivers: predictions requiring structural extrapolation from the training set receive higher nonconformity scores during calibration, producing wider intervals or larger prediction sets. This data-driven adaptation emerges without optimization; it is a mathematical consequence of using ECFP-based auxiliary models as nonconformity scores, which encode the same structural similarity intuition underlying AD assessment. Both take into account molecular similarity—AD checks similarity to training set chemicals; CP uses sigma models trained on ECFP of the training set to predict expected error and thus (partially) addresses the qualitative QSAR uncertainty described by Sahlin.^14^

The key distinction is formal: a high ADI score provides no guarantee of coverage, whereas CP empirical coverage matches the target level by construction. Moreover, conformal prediction is complementary to rather than a replacement for AD: existing AD metrics (leverage, Tanimoto nearest-neighbor distance, ensemble variance) can serve directly as nonconformity scores, embedding decades of AD research into a framework that converts heuristic similarity into statistically valid prediction intervals. The likelihood-based distance-to-model framework discussed in the Introduction provides a natural quantitative bridge to conformal prediction: likelihood scores that model p­(e|x) as a parametric function of structural distance can serve directly as nonconformity scores, combining the interpretability of DM methods with the formal guarantees of conformal calibration. Importantly, the coverage and efficiency metrics reported here—empirical coverage vs nominal level, and mean interval width or singleton rate across endpoints—already constitute the quantitative calibration diagnostics that allow assessment of whether a given nonconformity score, including the ECFP-based auxiliary models used here, is useful or whether simpler approximations suffice. The strong CP-ADI correlations observed across 100+ endpoints and millions of predictions,^36^ and the consistent achievement of nominal coverage, provide this evidence: structural fingerprint-based auxiliary models capture the dominant source of uncertainty without requiring parametric assumptions on the error distribution, while retaining the finite-sample guarantees that likelihood-based approaches lack.

The few endpoints showing weak CP efficiency—ADI correlation suggest that for some properties, prediction difficulty arises from factors beyond structural similarity alone, and investigation of appropriate nonconformity scores could be a direction for future work.

### 4.4 Ensemble and Weight-of-Evidence Extensions

Model ensembles: CP is a natural framework for ensemble prediction—by calibrating once on combined model outputs;^39^ aggregated conformal prediction using median interval bounds,^25^ or aggregate using reciprocal interval weights, which is akin to decision-making based on the weight of evidence. The latter approach was used by the authors to combine predictions of several models of the same property (e.g., 3 BCF models, etc.) within the context of predicting properties of chemical substances and novel polymers for SSbD assessment.^40^

### 4.5 Regulatory and SSbD Applications

For regulatory users, a conformal prediction interval communicates not only the likely outcome but also the degree of confidence, supporting more informed judgments about data sufficiency and the need for experimental follow-up. Within the SSbD context, CP enables early-stage assessments that explicitly acknowledge uncertainty, helping innovators identify promising candidates while flagging cases where experimental confirmation is essential. The prediction intervals can be directly used in multicriteria decision analysis models like SMAA.^41^ The framework supports fully automated batch uncertainty quantification—requiring no manual weight-of-evidence assessment—while remaining applicable to any QSAR/QSPR model or platform through the open-source pipeline provided. For QSAR practitioners, CP offers a direct way to compare models, assess prediction reliability across chemical series, and prioritize further testing, where uncertainty is greatest.

### 4.6 Limitations

Conformal prediction provides marginal coverage guarantees: the nominal coverage (e.g., 90%) holds in expectation across the full test set but is not guaranteed within chemical subgroups, structural classes, or individual molecules. A model may simultaneously overcover drug-like compounds and undercover environmental contaminants while reporting valid overall statistics. Recent diagnostics for detecting marginal coverage failures are available and recommended when subgroup validity is critical.

The combination of auxiliary-based pseudoprobabilities and predefined (likely nonexchangeable) train/test splits presents a realistic but challenging scenario for conformal prediction. In practice, new chemicals often differ systematically from training data, violating the exchangeability assumptions. Our results suggest that standard conformal methods may be overly conservative in such settings, achieving a valid but inefficient coverage. Future work should explore conformal methods robust to distribution shift, such as weighted conformal prediction^42^ or other recent domain-adaptive approaches. This is also recommended by a recent review.^25^

The current implementation produces symmetric prediction intervals of the form *ŷ* ± *q̂*·σ̂(*x*), which assume that prediction errors are distributed symmetrically around the point estimate. When the underlying model exhibits directional bias—for instance, systematically underpredicting highly active compounds—a symmetric interval will undercover one tail and overcover the other, even while satisfying the marginal guarantee overall. The principled remedy is asymmetric conformal prediction, where separate calibration quantiles are computed independently for the upper and lower residual tails; we identify this as a direction for future work.

## 5 Conclusions

This study demonstrates the potential of adaptive conformal prediction to move QSAR methodology beyond traditional, heuristic applicability domain (AD) proxies toward more rigorous interval/set-based uncertainty quantification. The core message of this work is not the specific auxiliary models or fingerprint choices demonstrated here but the framework itself: any QSAR model—regression or classification, legacy or newly developed—can be converted into a confidence predictor that reports prediction intervals or prediction sets with guaranteed marginal coverage at a user-specified confidence level. The efficiency metrics (interval width and set size) can be used to compare and interpret model outputs instead of the wide variety of AD metrics currently in place. The existing AD metrics are largely suitable for use as nonconformity scores.

For new model development, CP should be integrated from the outset rather than applied post hoc. A calibration set should be explicitly reserved during model construction, the nonconformity score selected alongside the model architecture, and empirical coverage and interval width reported as standard validation metrics alongside *R* ^2^ and RMSE for regression and alongside accuracy and AUC for classification. This requires minimal additional effort: mature, well-documented open-source libraries - including MAPIE,^21^ CREPES,^43,44^ https://github.com/ml-stat-Sustech/TorchCP, https://github.com/ryantibs/conformal, and https://github.com/donlnz/nonconformist - implement the full CP workflow and integrate directly with standard machine learning pipelines. For legacy models where retraining is not feasible, the posthoc approach demonstrated here—training an auxiliary model on calibration residuals using molecular structure—provides a practical entry point.

A broader recommendation emerges from the classification results: QSAR classification models should expose class probabilities rather than hard labels. The ordinal distance strategy introduced here demonstrates that pseudoprobabilities can be recovered from hard-label outputs, enabling conformal prediction sets with reasonable efficiency; however, this is a workaround rather than an ideal solution. Models that natively output calibrated class probabilities allow the direct application of standard conformal classification methods, yielding smaller prediction sets and more interpretable uncertainty estimates. Model developers and platform maintainers are encouraged to expose probability outputs as a default, supporting not only conformal prediction but also the overall goal of transparent uncertainty communication.

The framework is applicable to any QSAR or QSPR platform, and the strong alignment between conformal efficiency metrics and existing applicability domain indices demonstrated across 100+ endpoints suggests that adoption need not displace existing AD workflows—rather, CP provides the statistical calibration layer that converts AD intuitions into formally guaranteed uncertainty statements. Coverage and efficiency, reported alongside point and interval/set predictions, should become standard outputs of QSAR model validation and deployment, supporting more informed regulatory decision-making and accelerating the transition toward uncertainty-aware chemical safety assessment.

Finally, uncertainty-aware reasoning is already standard practice in risk assessment: experimental measurements are routinely reported with uncertainty bounds, and regulatory decisions accommodate uncertainty factors. Conformal prediction simply extends this established practice to computational predictions, making QSAR outputs more directly comparable to experimental data that they are designed to complement.

## Supplementary Material



## Notes

Archive with figures, .png files of figures, and data files with statistics used to create the figures (ZIP); A separate archive with Excel files with conformal prediction for VEGA training and test data sets and 500000 EPA CompTox compounds is uploaded to Zenodo^36^ (ZIP)

## Abbreviations

CP: conformal prediction
ADI: applicability domain index
LAC: Least Ambiguous Set
APS: Adaptive Prediction Sets
MAPIE: Model Agnostic Prediction Interval Estimator
PARC: Partnership for the Assessment of Risks from Chemicals
