# Supplementary material for: Beyond Heuristics: A Model-Agnostic Framework for Uncertainty Quantification in QSAR via Adaptive Conformal Prediction
Source: Chem Res Toxicol. 2026 Jun 22;39(7):1357–76. doi: 10.1021/acs.chemrestox.6c00065 (PMC13390030; doi:10.1021/acs.chemrestox.6c00065)
Supplement: Supplementary file 1 [file tx6c00065_si_001.zip › SupportingInfo_conformal_prediction_crt_specialissue_NAM_jeliazkova/cover_page.docx]

Supporting information files

Beyond Heuristics: A Model-Agnostic Framework for Uncertainty Quantification in QSAR via Adaptive Conformal Prediction

Nina Jeliazkova*, Nikolay Kochev, Luchesar Iliev , Vedrin Jeliazkov

Ideaconsult Ltd. Sofia 1000, Bulgaria.

*corresponding author email: [jeliazkova.nina@gmail.com](mailto:jeliazkova.nina@gmail.com)

The Supporting Information contains

# Supporting_info.doc – examples and illustrations on

- 1. Distance-to-model and conformal efficiency metrics
  2. Exchangeability tests examples and illustrations
  3. Overfitting / underfitting auxiliary (sigma) models considerations

# Figures and summary statistics

The original high-resolution figures from the main manuscript together with associated summary statistics, uncertainty domain analyses, coverage efficiency evaluations, correlation analyses, and supplementary dataset tables for both classification and regression studies. The full datasets are available at Zenodo ^1^.

**2.1. Classification (LAC)**

**2.1.1. VEGA Datasets**

- Coverage efficiency analysis (coverage_efficiency_analysis-1.xlsx)
- Figure 5. NCM comparison for classification (Fig05.ncm_comparison_classification.png)
- Figure 11. Coverage efficiency (Fig11.coverage_efficiency-1.png)
- Figure 12. ADI bins distance analysis (Fig12.adi_bins_distance-1.png)
- Figure 13. Spearman correlation analysis (Fig13.spearman-1.png)
- Spearman correlation data (spearman-1.xlsx)
- Dataset summary (summary.xlsx)

**2.1.2. CompTox Datasets**

- Figure 14. Uncertainty domain figure for global models (Fig14. uncertainty_domain_figure_global_models.png)
- Global model uncertainty statistics (uncertainty_domain_figure_global_global_stats.csv)
- ADI matrix for global models (uncertainty_domain_figure_global_model_adi_matrix.csv)
- Summary of global uncertainty domain analysis (uncertainty_domain_figure_global_summary.txt)

**2.2. Regression**

**2.2.1. Vega Datasets**

- Coverage efficiency results (coverage_efficiency-2.xlsx)
- Figure 3. Regression NCM comparison (Fig03.regression_ncm_comparison.png)
- Figure 4. Regression dataset difficulty analysis (Fig04.regression_dataset_difficulty_all.png)
- Figure 6. Coverage efficiency (Fig06.coverage_efficiency-2.png)
- Figure 7. Coverage analysis (Fig07.coverage_analysis-2.png)
- Figure 8. Spearman correlation analysis (Fig08.spearman-2.png)
- Figure 2. BCF MEYLAN HENRY OPERA scatter plot (Figure 2. BCF_MEYLAN_HENRY_OPERA_scatter.png)
- Figure 15 (Figure15.png)
- Spearman correlation data (spearman-2.xlsx)
- Dataset summary (summary.xlsx)

**2.2.2. CompTox Datasets**

- Figure 9. Uncertainty domain figure (global) (Fig09.uncertainty_domain_figure_global.png)
- Figure 10. Uncertainty domain figure for global models (Fig10.uncertainty_domain_figure_global_models.png)
- Global model uncertainty statistics (uncertainty_domain_figure_global_global_stats.csv)
- ADI matrix for global models (uncertainty_domain_figure_global_model_adi_matrix.csv)
- Summary of global uncertainty domain analysis (uncertainty_domain_figure_global_summary.txt)

# References

(1) Jeliazkova, N.; Kochev, N.; Iliev, L.; Jeliazkov, V. Uncertainty-Quantified QSAR Predictions: VEGA Models (98 Endpoints) with Conformal Intervals/Sets for Training, Test, and EPA CompTox Datasets (500K Compounds). Zenodo 2026. https://doi.org/10.5281/zenodo.18444068.
