# Supplementary material for: Beyond Heuristics: A Model-Agnostic Framework for Uncertainty Quantification in QSAR via Adaptive Conformal Prediction
Source: Chem Res Toxicol. 2026 Jun 22;39(7):1357–76. doi: 10.1021/acs.chemrestox.6c00065 (PMC13390030; doi:10.1021/acs.chemrestox.6c00065)
Supplement: Supplementary file 1 [file tx6c00065_si_001.zip › SupportingInfo_conformal_prediction_crt_specialissue_NAM_jeliazkova/Supporting_info.docx]

# Distance-to-model and conformal efficiency metrics

To determine which is the better Applicability Domain (AD) measure for the different distance-to-model indices, we have to look at how well each index correlates with the conformal efficiency (the interval width). In a Conformal Prediction (CP) framework, a "good" AD measure is one that effectively flags high-uncertainty compounds by assigning them wider intervals.

The calculatons and figures are performed with the tutorial pipeline at <https://github.com/ideaconsult/qubounds>

ASNN-STDEV (Figure S1) is the significantly better AD measure compared to ASNN-CORREL (Figure S2), as indicated in the table:

| Metric | ASNN-STDEV | ASNN-CORREL |
| --- | --- | --- |
| Spearman Correlation (ρ) with interval width | 0.333 | 0.248 |
| Trend Direction | Positive (Higher STDEV = Wider) | Positive (Higher CORREL = Wider) |
| In-AD vs. Out-of-AD Separation | Significant (p < 0.0000) | Less Significant (p = 0.0220) |
| Mean Width Shift | 1.73 → 2.49 (Stronger) | 2.33 → 1.80 (Unexpected Inverse) |
| Sample Distribution | Balanced | Highly Skewed |

**
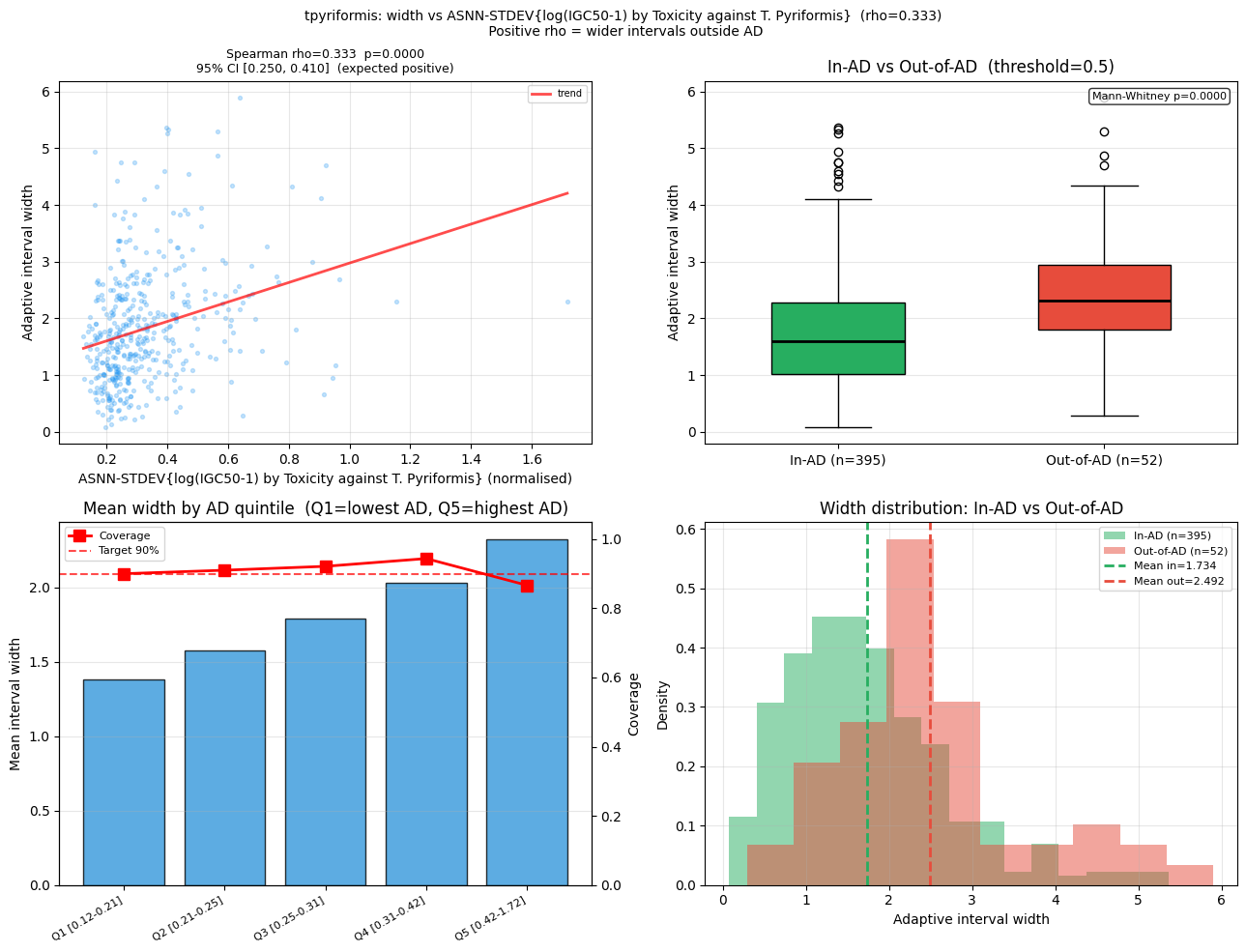
**

Figure S1. Relationship between adaptive conformal interval width and applicability domain for the T. pyriformis toxicity regression model. * Top left: Scatter plot of adaptive prediction interval width versus the normalized ASNN-STDEV. The positive Spearman correlation demonstrates that the framework adaptively generates wider intervals for compounds further from the training domain.Top right: Box plot comparing interval widths for compounds categorized as "In-Domain" (green, ASNN-STDEV < 0.5) versus "Out-of-Domain" (red, ASNN-STDEV >e 0.5). The Mann-Whitney test confirms a statistically significant difference, with out-of-domain compounds receiving more conservative (wider) uncertainty estimates.Bottom left: Mean interval width stratified by AD quintiles (Q1 to Q5). The overlaying red line indicates the empirical coverage, which remains consistently near the 90% target level across all quintiles, illustrating that CP maintains the statistical guarantee regardless of structural novelty.Bottom right: Density distribution of interval widths for In-AD vs. Out-of-AD compounds, showing a clear shift in the mean, highlighting the framework's ability to discriminate uncertainty based on chemical similarity.


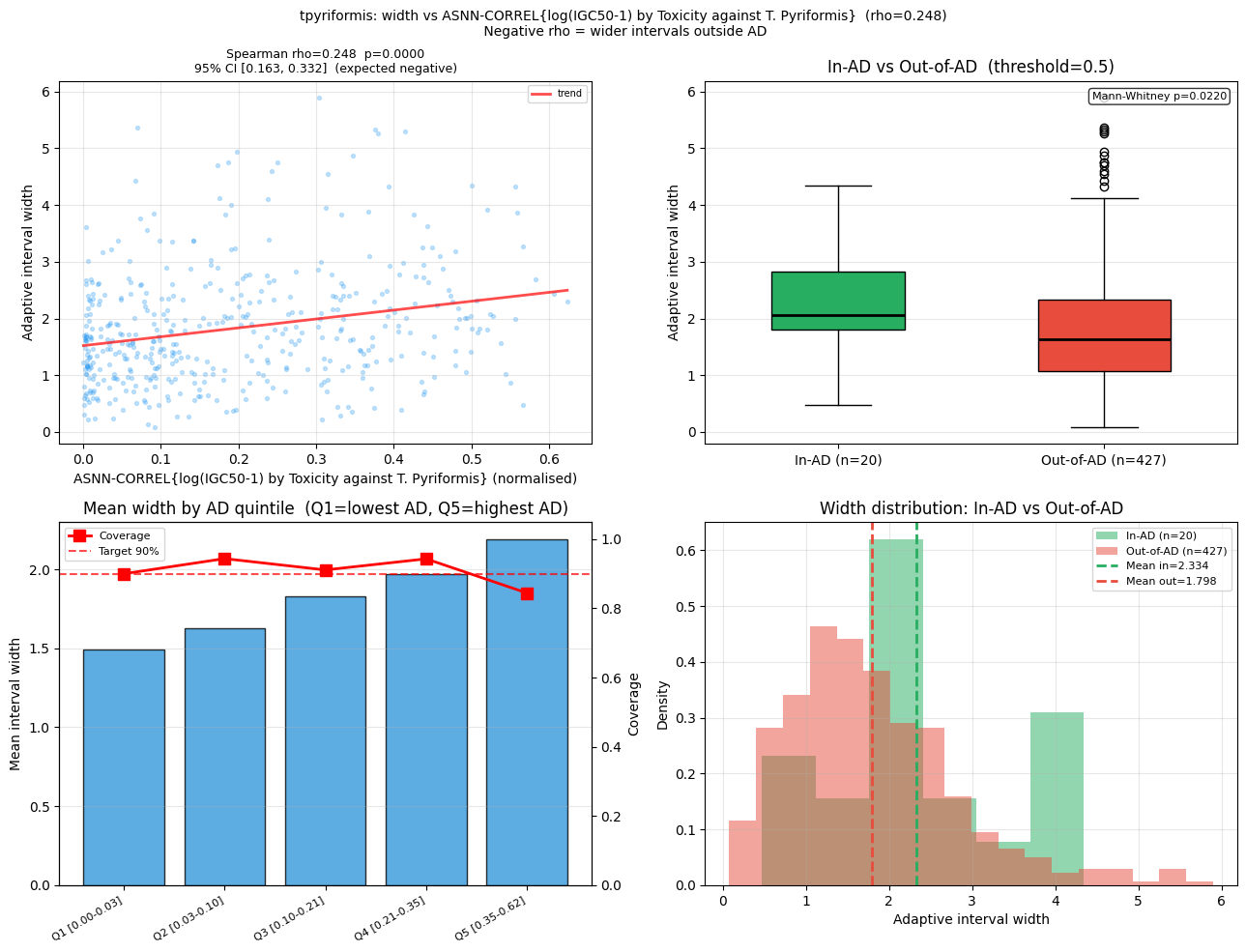


Figure S2. Relationship between adaptive conformal interval width and applicability domain for the T. pyriformis toxicity regression model. * Top left: Scatter plot of adaptive prediction interval width versus the normalized ASNN-CORREL. The positive Spearman correlation demonstrates that the framework adaptively generates wider intervals for compounds further from the training domain.Top right: Box plot comparing interval widths for compounds categorized as "In-Domain" (green, ASNN-CORREL < 0.5) versus "Out-of-Domain" (red, ASNN-CORREL > 0.5). The Mann-Whitney test confirms a statistically significant difference, with out-of-domain compounds receiving more conservative (wider) uncertainty estimates.Bottom left: Mean interval width stratified by ASNN-CORREL quintiles (Q1 to Q5). The overlaying red line indicates the empirical coverage, which remains consistently near the 90% target level across all quintiles, illustrating that CP maintains the statistical guarantee regardless of structural novelty.Bottom right: Density distribution of interval widths for In-AD vs. Out-of-AD compounds, showing a clear shift in the mean, highlighting the framework's ability to discriminate uncertainty based on chemical similarity

# Exchangeability tests examples

The coverage guarantee holds under the exchangeability assumption: calibration and test compounds are drawn from the same input space. When this is violated (structural extrapolation), coverage may fall below the nominal level. The pipeline signals this through:

- widening prediction intervals or shrinking singleton rates for out-of-domain compounds,
- Kolmogorov-Smirnov tests comparing calibration and test nonconformity score distributions,
- conformal p-value uniformity tests (p-values should be uniform under exchangeability).

These diagnostics are computed automatically in all tutorial and full pipeline tasks <https://github.com/ideaconsult/qubounds>


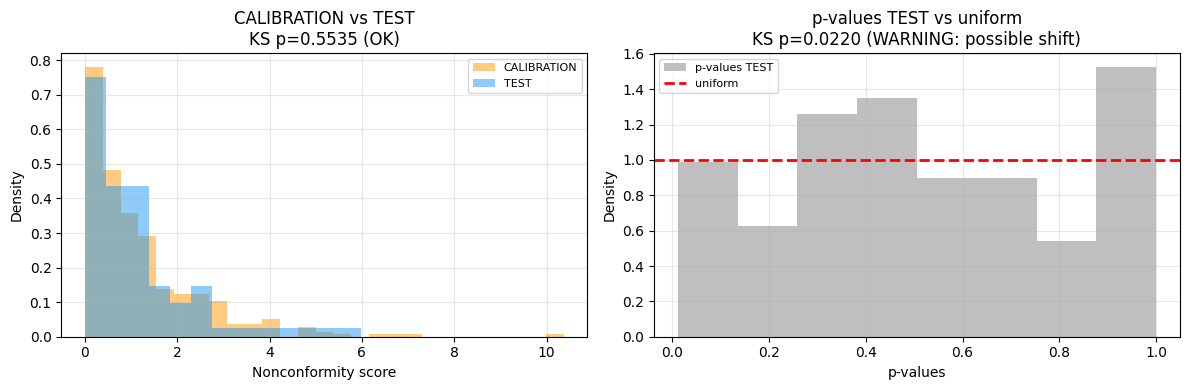


Figure S3. T.pyriformis regression model exchangeability tests. Generated with tutorial pipeline at https://github.com/ideaconsult/qubounds


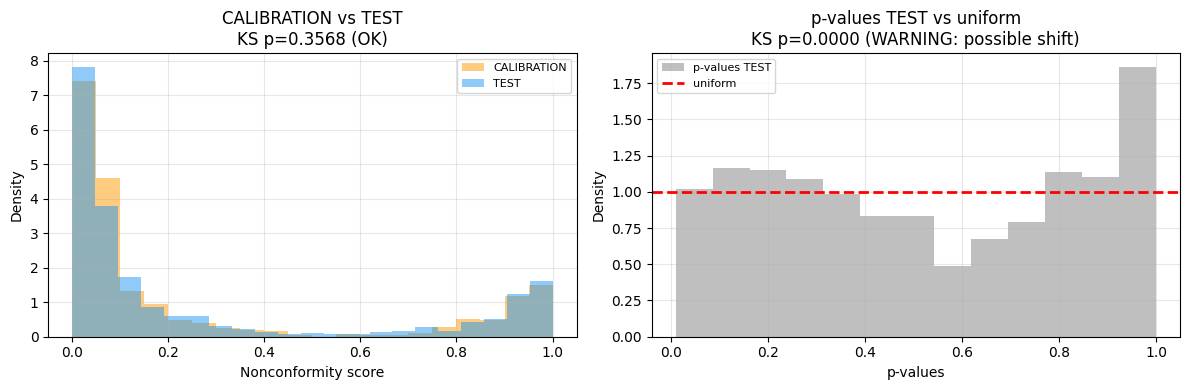


Figure S4. Ames mutagenicity classification model exchangeability tests. Generated with tutorial pipeline at https://github.com/ideaconsult/qubounds

# Overfitting / underfitting auxiliary (sigma) models considerations

The sigma models are trained to predict the absolute residual of the base model from ECFP fingerprints. This is an inherently noisy target: the residual for any individual compound reflects not only structural features that make it genuinely difficult to predict, but also experimental measurement noise. The practical consequence is that the sigma model provides weaker normalization than an ideal uncertainty estimator would, and the resulting CP intervals are closer to those of standard non-normalized conformal prediction. This is a conservative failure mode: the marginal coverage guarantee is preserved, but the intervals are less efficient - they may be unnecessarily wide for well-covered compounds and insufficiently narrow for genuinely difficult ones. However the actual sigma model quality is not as critical as for the base model, because the output of sigma model (the nonconformity score) are only used for ranking and quantile calculation (Figure S5).


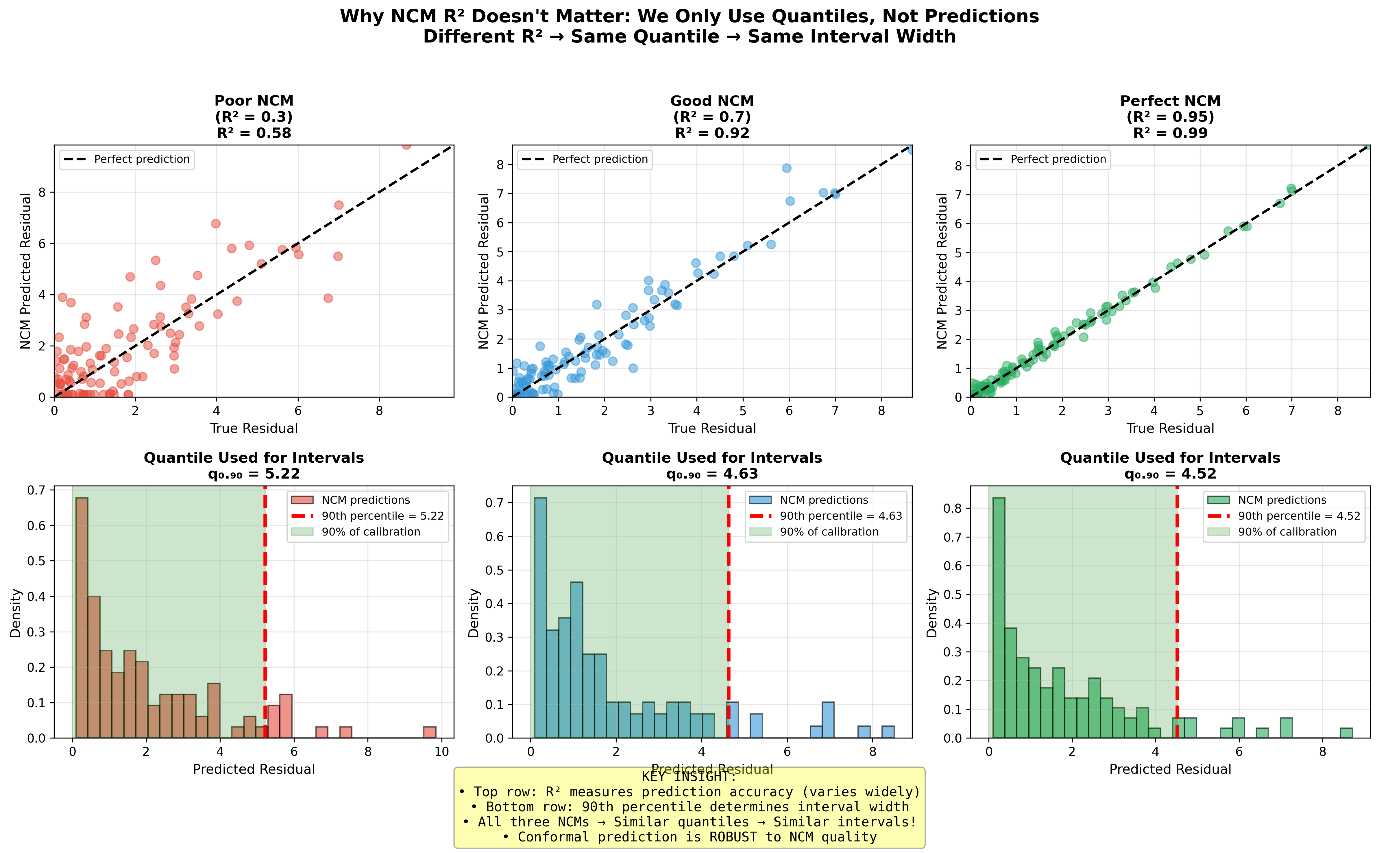


Figure S5. Illustration of the robustness of Conformal Prediction - the quality of a Non-Conformity Measure (NCM) has a surprisingly small impact on the final prediction interval width. Simulated data.

True overfitting of the sigma model - in which specific training compound residuals are memorized and projected onto new compounds - is less likely given the noise level of the target variable and the regularization implicit in standard learners. However, we do see overfitting in kNN auxiliary models and intentionally selected in both regression and classification case gradient boosting methods instead, which are known to have better regularization capabilities.

References

(1) Jeliazkova, N.; Kochev, N.; Iliev, L.; Jeliazkov, V. Uncertainty-Quantified QSAR Predictions: VEGA Models (98 Endpoints) with Conformal Intervals/Sets for Training, Test, and EPA CompTox Datasets (500K Compounds). Zenodo 2026. https://doi.org/10.5281/zenodo.18444068.
