# Supplementary figures and images for: Beyond Heuristics: A Model-Agnostic Framework for Uncertainty Quantification in QSAR via Adaptive Conformal Prediction
Source: Chem Res Toxicol. 2026 Jun 22;39(7):1357–76. doi: 10.1021/acs.chemrestox.6c00065 (PMC13390030; doi:10.1021/acs.chemrestox.6c00065)

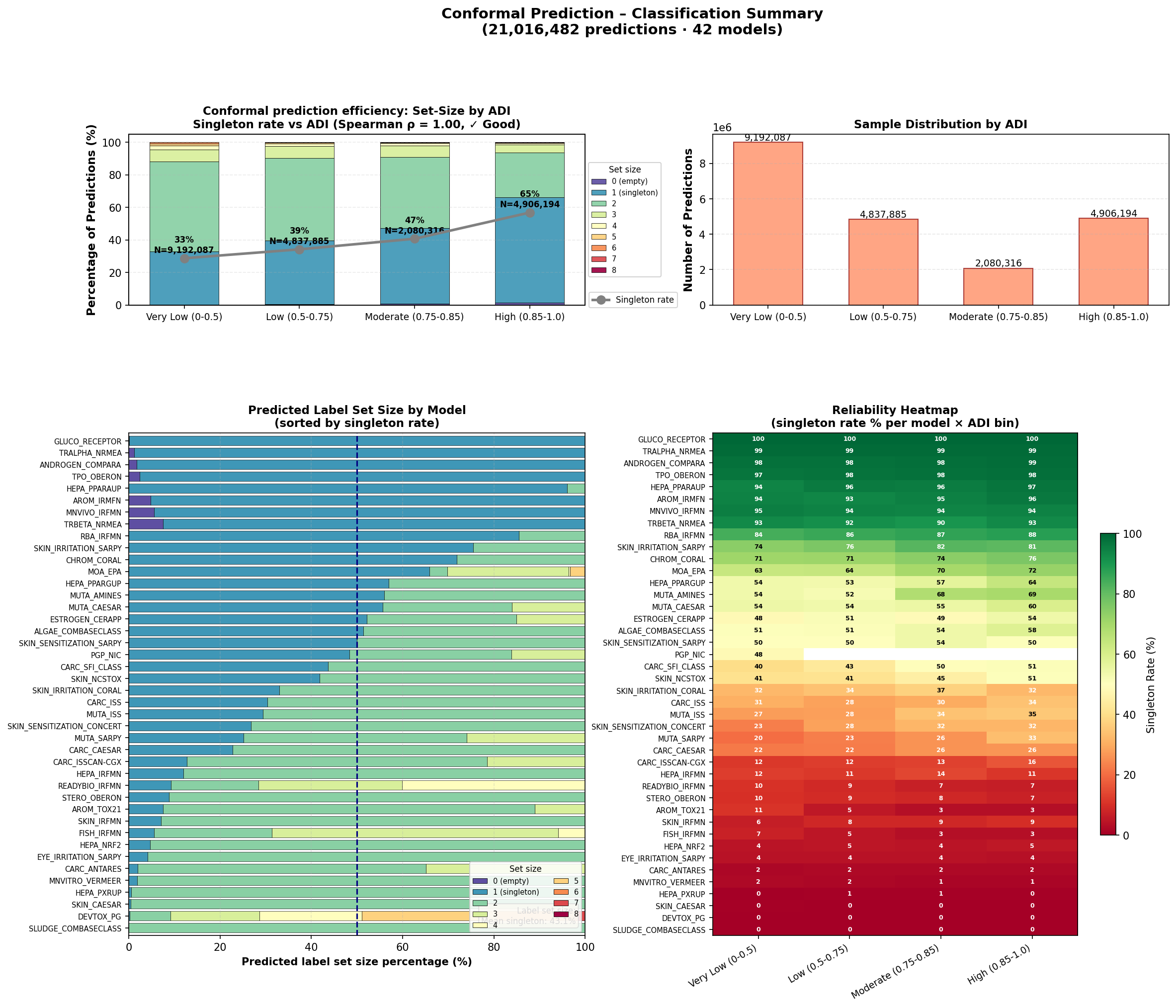

Supplement: Supplementary file 1 [file tx6c00065_si_001.zip › SupportingInfo_conformal_prediction_crt_specialissue_NAM_jeliazkova/class_lac/comptox/Fig14. uncertainty_domain_figure_global_models.png]

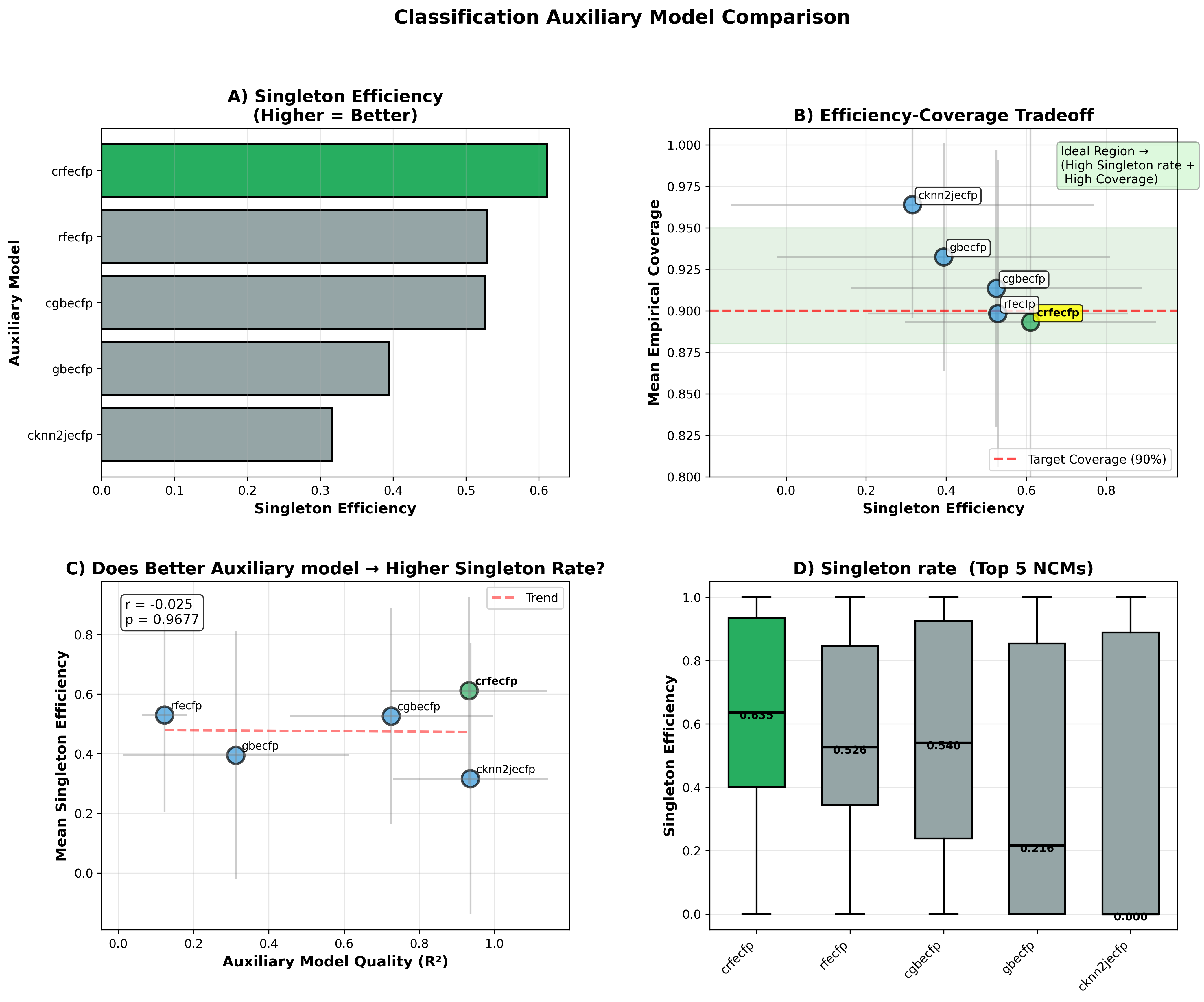

Supplement: Supplementary file 1 [file tx6c00065_si_001.zip › SupportingInfo_conformal_prediction_crt_specialissue_NAM_jeliazkova/class_lac/vega_datasets/Fig05.ncm_comparison_classification.png]

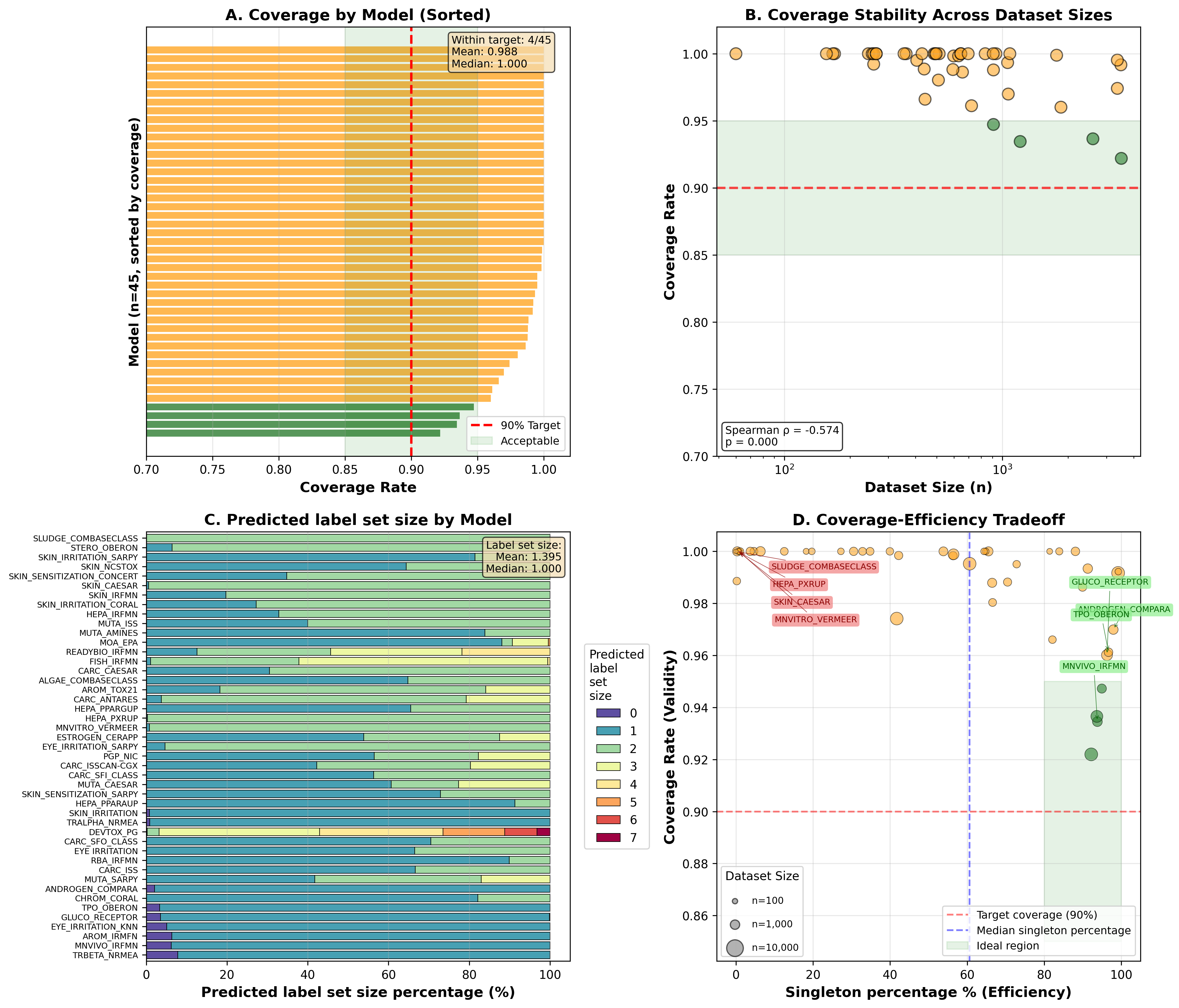

Supplement: Supplementary file 1 [file tx6c00065_si_001.zip › SupportingInfo_conformal_prediction_crt_specialissue_NAM_jeliazkova/class_lac/vega_datasets/Fig11.coverage_efficiency-1.png]

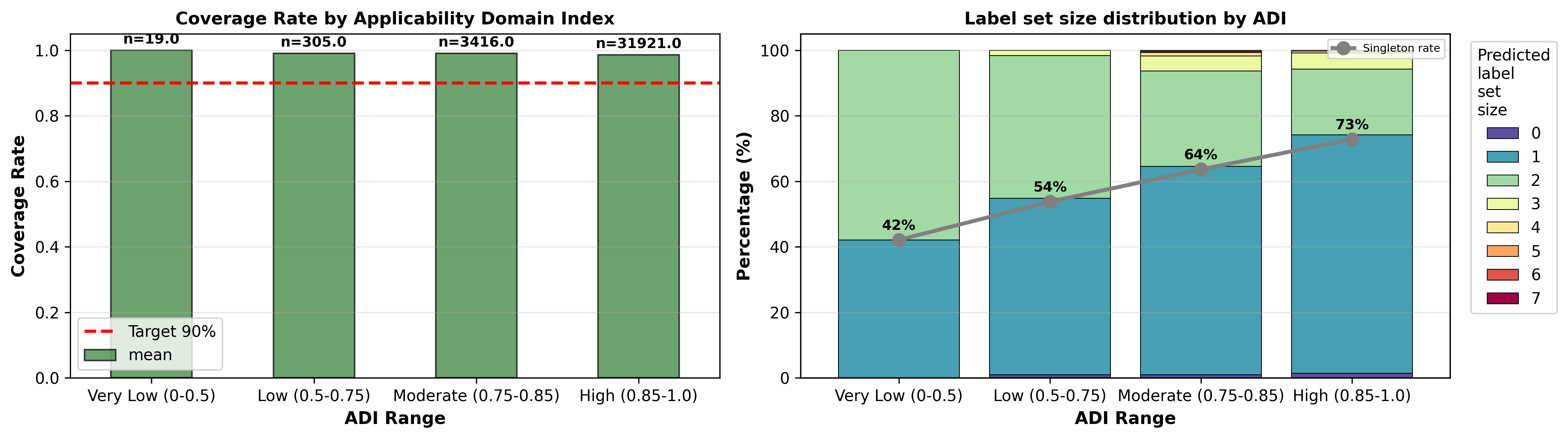

Supplement: Supplementary file 1 [file tx6c00065_si_001.zip › SupportingInfo_conformal_prediction_crt_specialissue_NAM_jeliazkova/class_lac/vega_datasets/Fig12.adi_bins_distance-1.png]

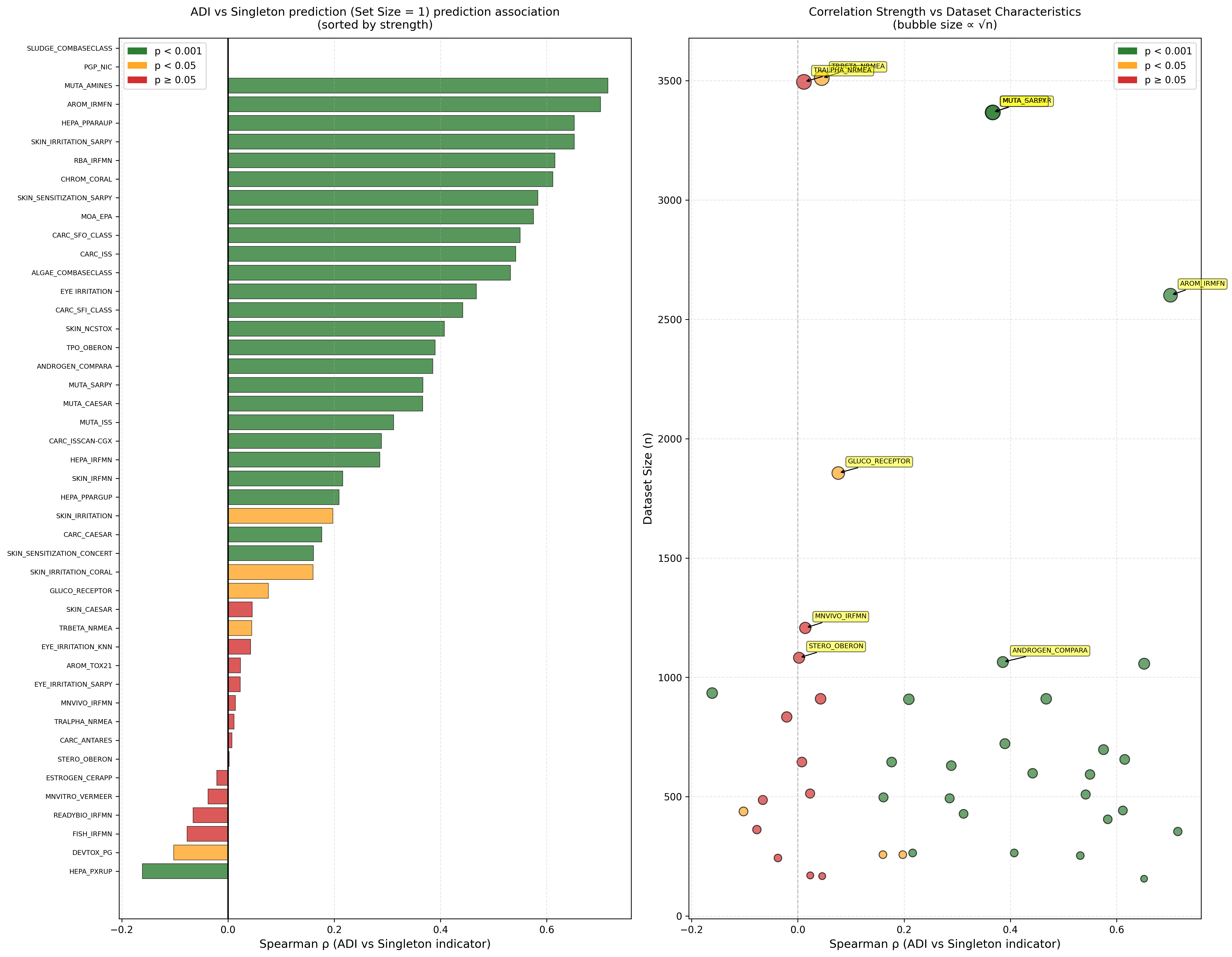

Supplement: Supplementary file 1 [file tx6c00065_si_001.zip › SupportingInfo_conformal_prediction_crt_specialissue_NAM_jeliazkova/class_lac/vega_datasets/Fig13.spearman-1.png]

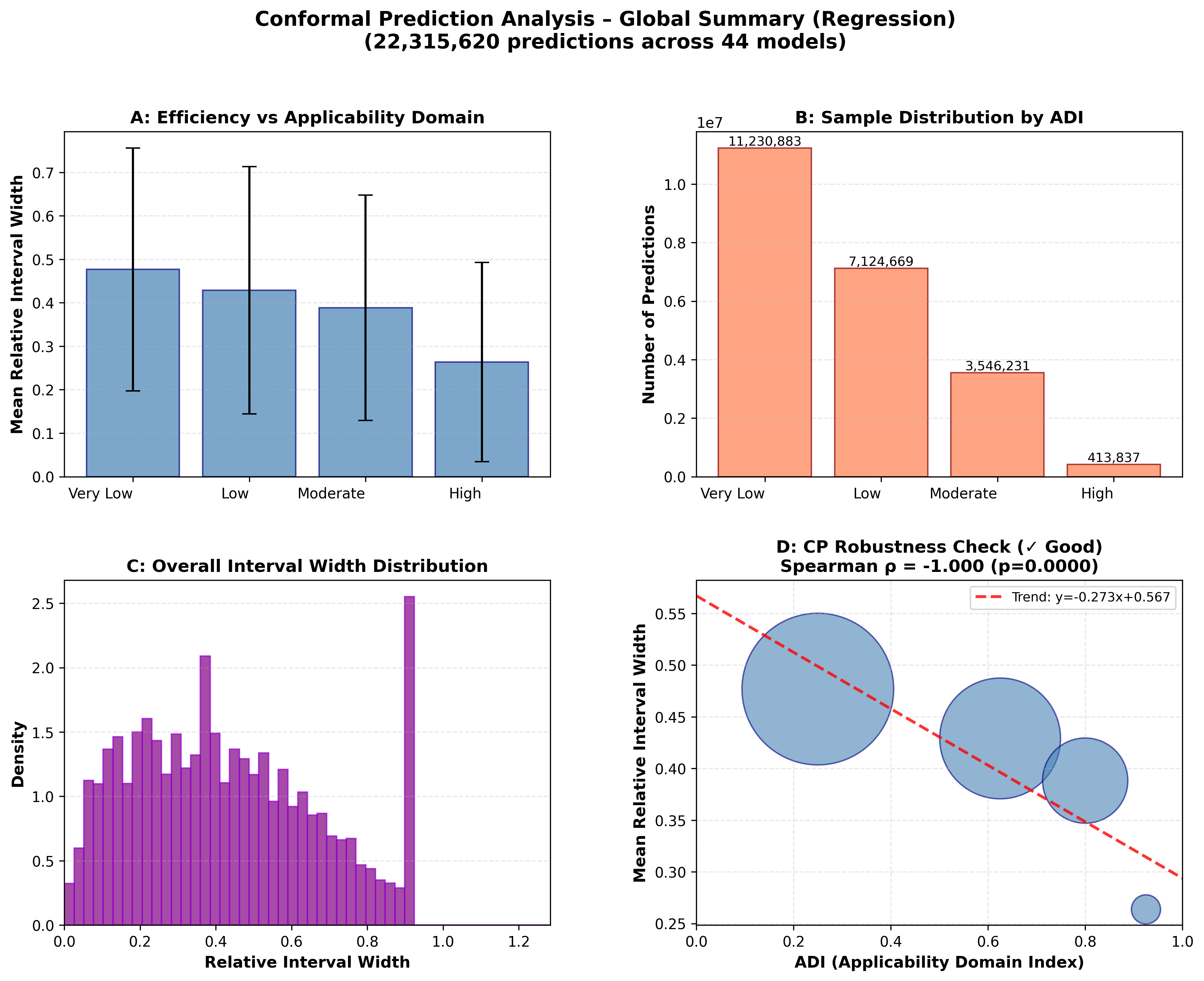

Supplement: Supplementary file 1 [file tx6c00065_si_001.zip › SupportingInfo_conformal_prediction_crt_specialissue_NAM_jeliazkova/regression/comptox/Fig09.uncertainty_domain_figure_global.png]

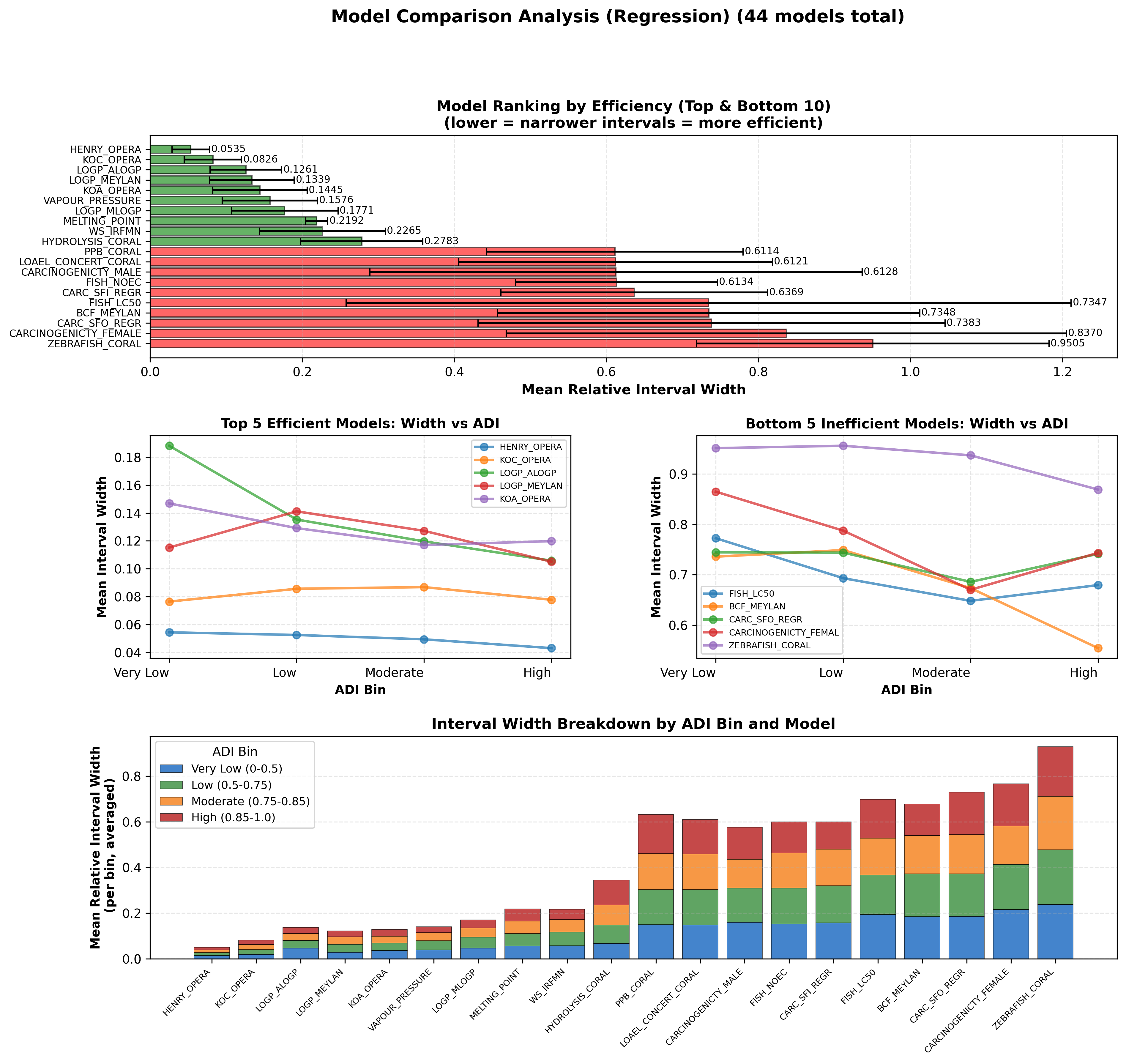

Supplement: Supplementary file 1 [file tx6c00065_si_001.zip › SupportingInfo_conformal_prediction_crt_specialissue_NAM_jeliazkova/regression/comptox/Fig10.uncertainty_domain_figure_global_models.png]

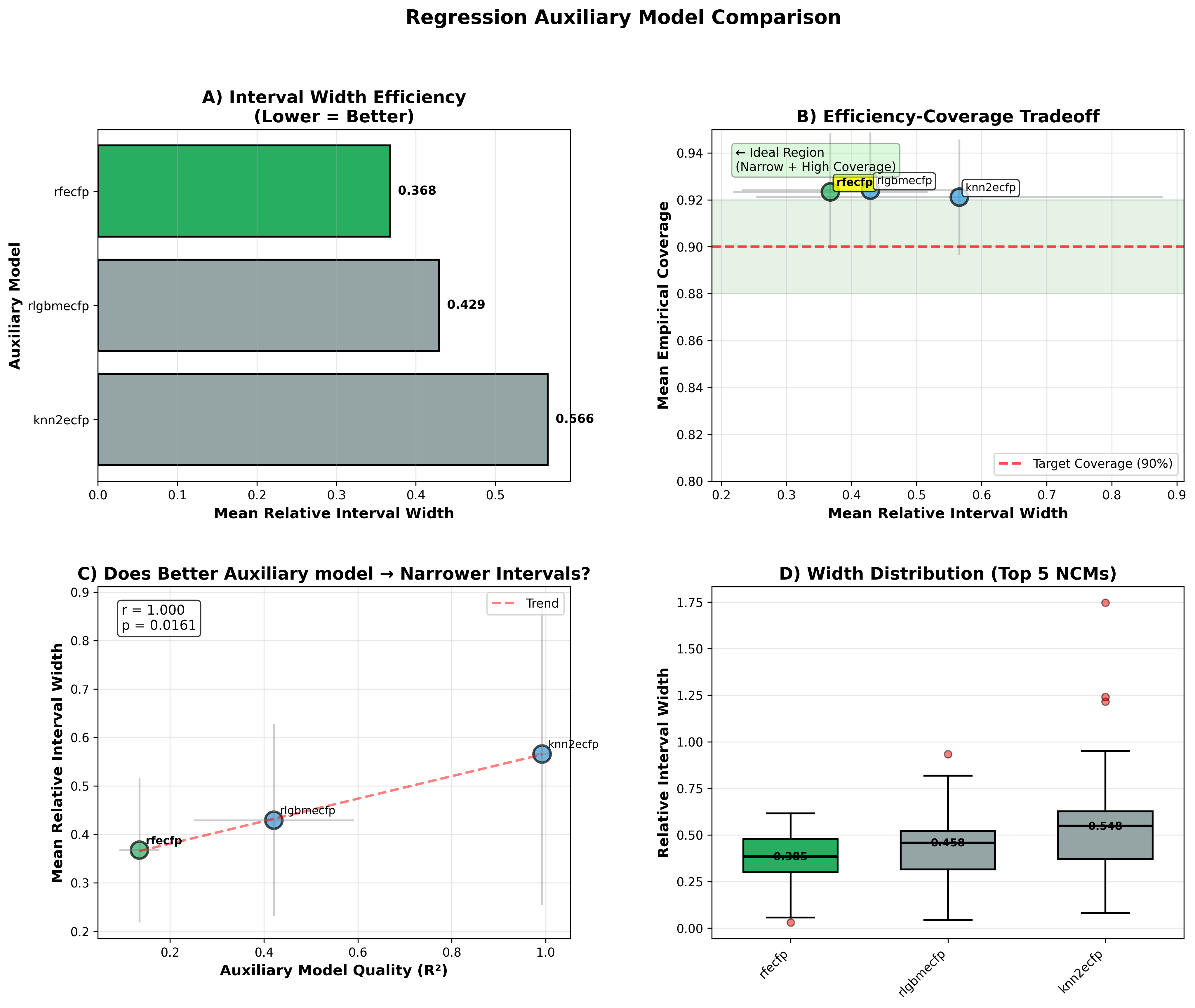

Supplement: Supplementary file 1 [file tx6c00065_si_001.zip › SupportingInfo_conformal_prediction_crt_specialissue_NAM_jeliazkova/regression/vega_datasets/Fig03.regression_ncm_comparison.png]

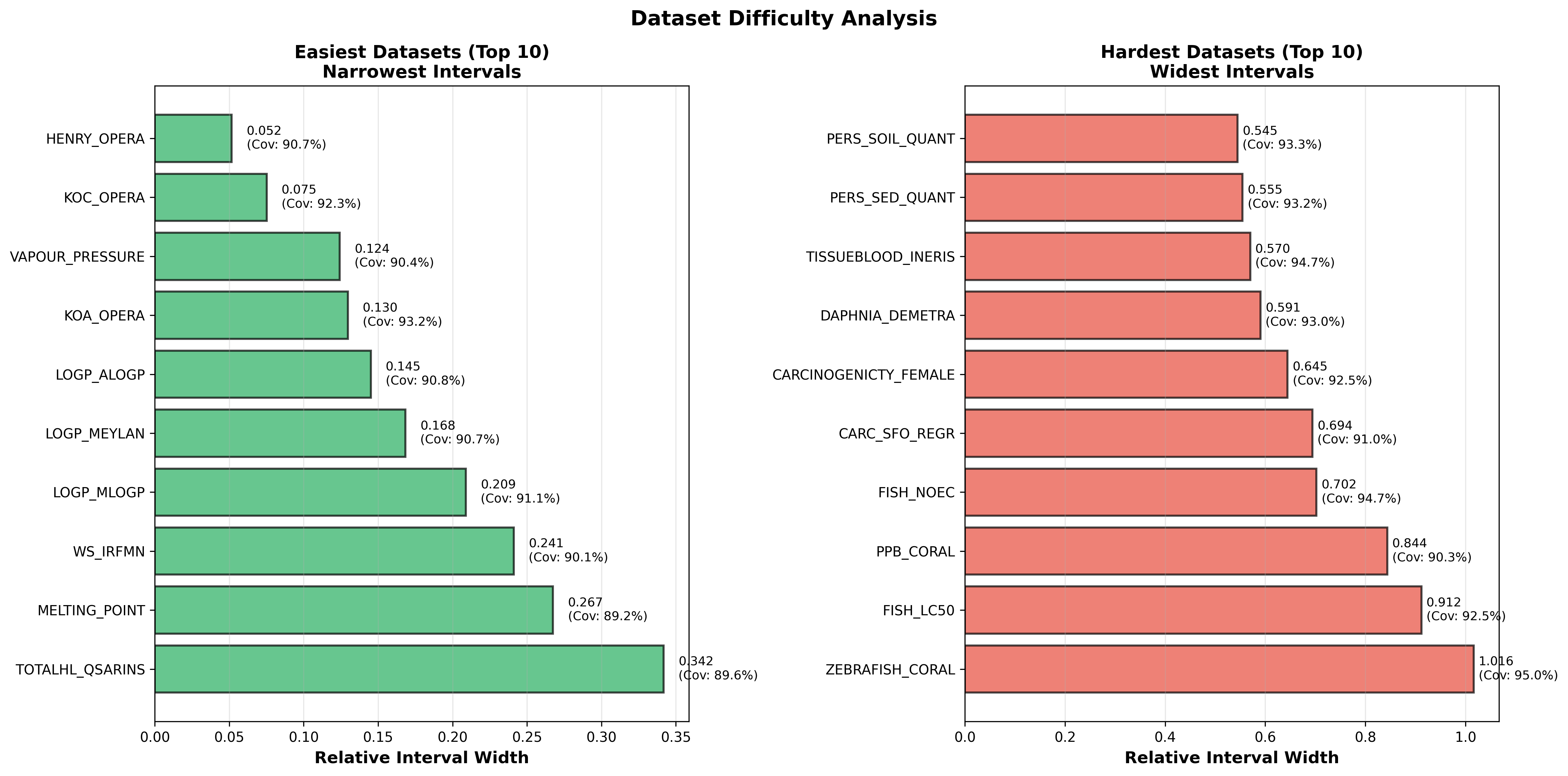

Supplement: Supplementary file 1 [file tx6c00065_si_001.zip › SupportingInfo_conformal_prediction_crt_specialissue_NAM_jeliazkova/regression/vega_datasets/Fig04.regression_dataset_difficulty_all.png]

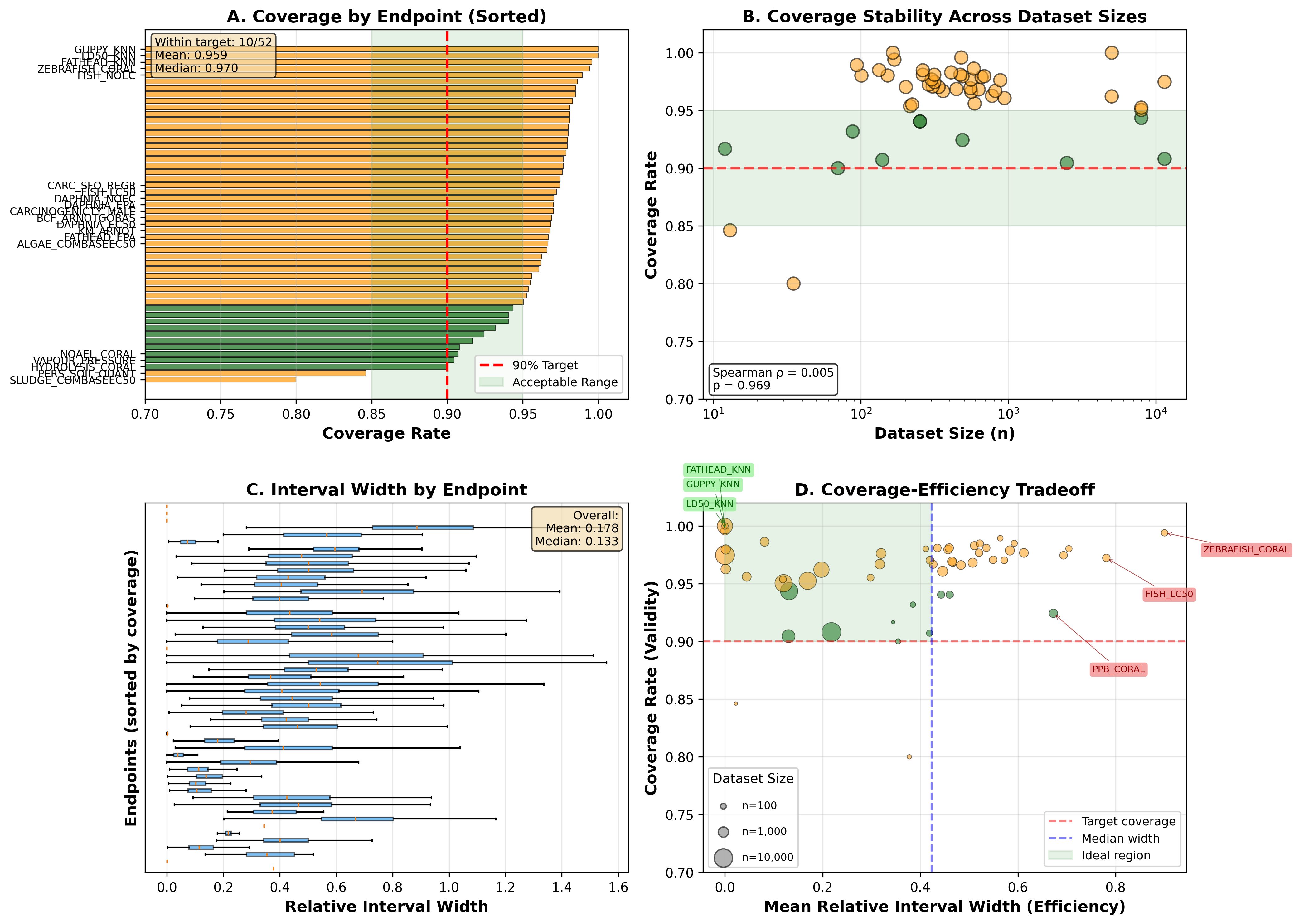

Supplement: Supplementary file 1 [file tx6c00065_si_001.zip › SupportingInfo_conformal_prediction_crt_specialissue_NAM_jeliazkova/regression/vega_datasets/Fig06.coverage_efficiency-2.png]

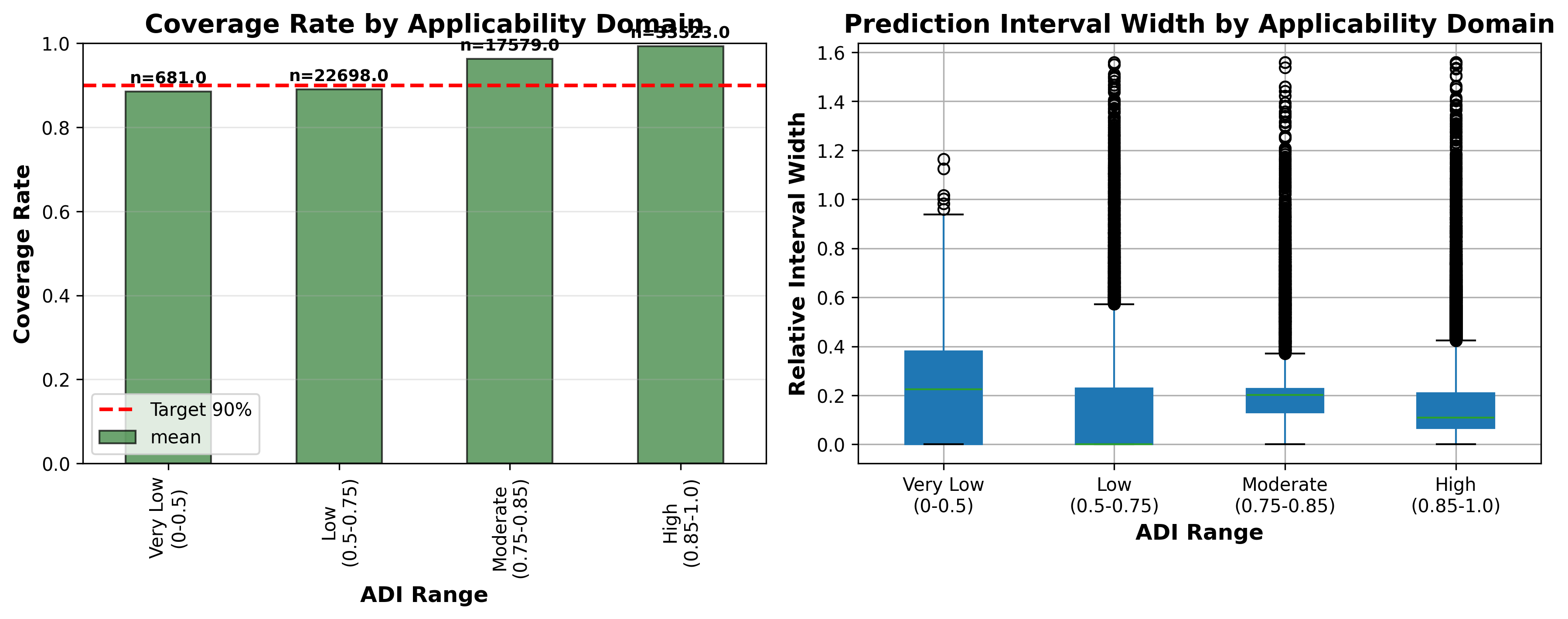

Supplement: Supplementary file 1 [file tx6c00065_si_001.zip › SupportingInfo_conformal_prediction_crt_specialissue_NAM_jeliazkova/regression/vega_datasets/Fig07.coverage_analysis-2.png]

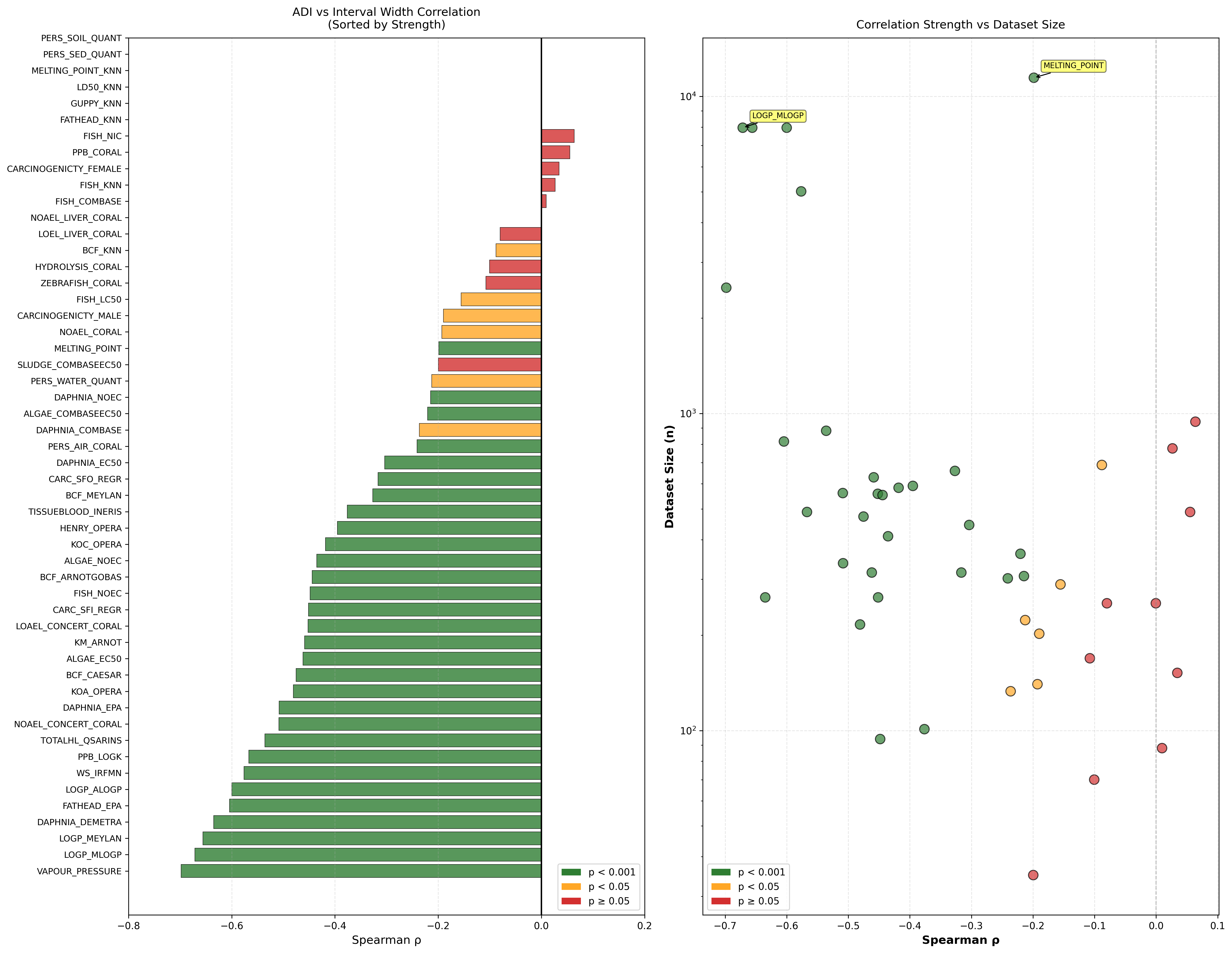

Supplement: Supplementary file 1 [file tx6c00065_si_001.zip › SupportingInfo_conformal_prediction_crt_specialissue_NAM_jeliazkova/regression/vega_datasets/Fig08.spearman-2.png]

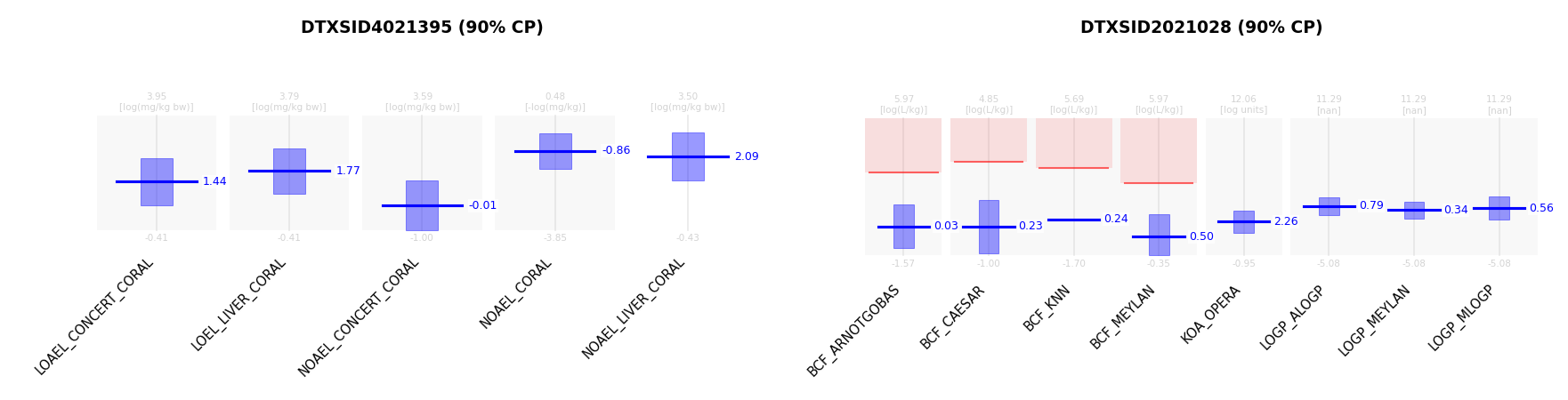

Supplement: Supplementary file 1 [file tx6c00065_si_001.zip › SupportingInfo_conformal_prediction_crt_specialissue_NAM_jeliazkova/regression/vega_datasets/Figure15.png]

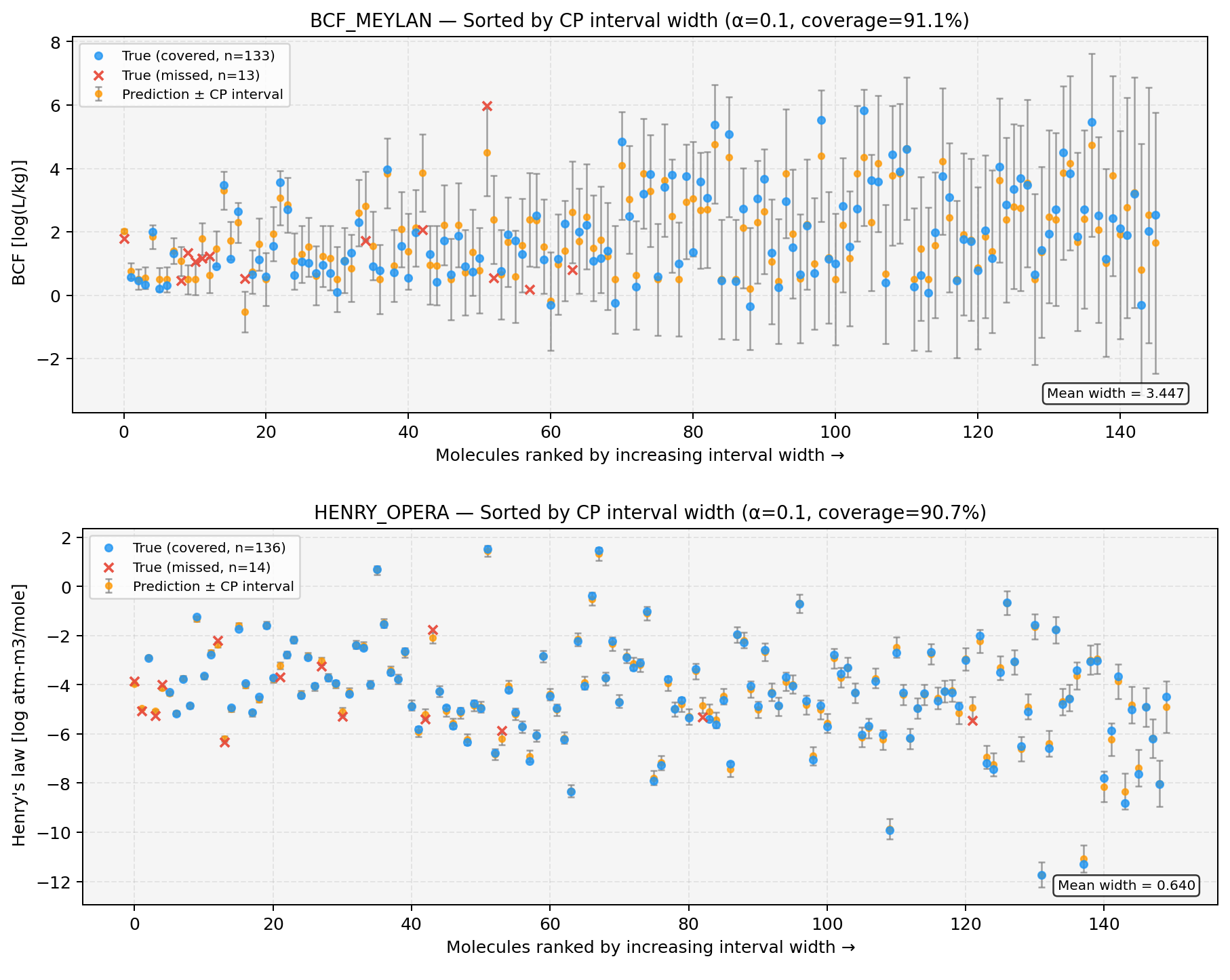

Supplement: Supplementary file 1 [file tx6c00065_si_001.zip › SupportingInfo_conformal_prediction_crt_specialissue_NAM_jeliazkova/regression/vega_datasets/Figure2.png]
